# A Chemiluminescence‐Activated Photodynamic Platform to Penetrate MRSA Biofilms and Counteract H_2_S‐Mediated Defense

**DOI:** 10.1002/smsc.202500618

**Published:** 2026-03-13

**Authors:** WeiYe Ren, WeiYi Cheng, Li He, JingQuan Chen, Liting He, Haorong Li, Guoying Zhou, Yinghui Wei, Ji‐Gang Piao, Dandan Bao

**Affiliations:** ^1^ School of Pharmaceutical Sciences The First Affiliated Hospital of Zhejiang Chinese Medical University Hangzhou China; ^2^ Hangzhou TCM Hospital Affiliated to Zhejiang Chinese Medical University Hangzhou China; ^3^ School of Molecular Medicine Hangzhou Institute for Advanced Study UCAS Hangzhou China; ^4^ School of Life Sciences of Zhejiang Chinese Medical University Hangzhou China; ^5^ Jinhua Academy of Zhejiang Chinese Medical University Jinhua China; ^6^ Department of Dermatology & Cosmetology The First Affiliated Hospital of Zhejiang Chinese Medical University Hangzhou China

**Keywords:** antibiofilm, chem‐stimulated antimicrobial photodynamic therapy, H_2_S self‐defense mechanism, methicillin‐resistant *Staphylococcus aureus*, penetration

## Abstract

The rapid escalation of antibiotic resistance has profoundly limited the effectiveness of conventional antimicrobial therapies, underscoring an urgent need for alternative antibacterial strategies. In this study, we present an innovative, antibiotic‐free dual‐action antibacterial platform designed to effectively eradicate recalcitrant methicillin‐resistant *Staphylococcus aureus* (MRSA) biofilm‐associated infections. Building upon conventional antimicrobial photodynamic therapy (APDT), a chemiluminescence‐based activation system is introduced to enable in situ APDT excitation, thereby overcoming the limited tissue penetration depth associated with external light sources. The byproduct CO_2_ gas not only disrupts the biofilm structure but also provides physical propulsion to drug‐loaded nanocarriers, facilitating the deep and uniform penetration of antimicrobial agents into the biofilm. Furthermore, hypericin functions not only as a photosensitizer that induces reactive oxygen species generation and oxidative stress in MRSA but also as an inhibitor of cystathionine β‐synthase (CBS), thereby disrupting the bacterial H_2_S‐based self‐defense pathway. Collectively, the in situ activation of photosensitizers via chemiluminescence represents a novel antibacterial strategy that overcomes the intrinsic limitations of light penetration. By enabling precise, localized activation of multiple antibacterial mechanisms, this approach offers a promising therapeutic solution for the treatment of recalcitrant MRSA infections and related diseases, with strong potential for clinical translation.

## Graphical abstract

A chemiluminescence‐activated photodynamic system is developed to eradicate methicillin‐resistant *Staphylococcus aureus* (MRSA) biofilms without external light or antibiotics. CO_2_ generated in situ disrupts biofilms and propels nanocarriers for deep penetration, while hypericin generates reactive oxygen species and suppresses H_2_S‐mediated defenses. This dual‐action strategy offers a potent and clinically promising solution for refractory MRSA infections.

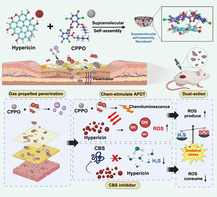

## 1 Introduction

Wound bacterial infections are a primary factor contributing to delayed wound healing, imposing significant physiological and psychological burdens on patients and placing substantial economic strain on healthcare systems [1]. The widespread presence of bacterial biofilms (BFs) in chronic wounds is a critical factor contributing to the difficulty of infection eradication [2]. Biofilms represent a specialized mode of bacterial organization, in which microorganisms adapt to their local microenvironment by secreting extracellular polymeric substances (EPS) that assemble into highly organized, dense three‐dimensional structures [3, 4, 5]. This architecture not only shields bacteria from host immune responses but also effectively blocks the penetration and diffusion of antimicrobial agents, making it challenging for these agents to reach the active bacterial populations deep within the biofilm, thereby compromising the efficacy of conventional antimicrobial therapies [6, 7, 8]. Of particular concern is the formation of heterogeneous microenvironments within biofilms, driven by nutrient limitation, pH gradients, and reduced oxygen availability. These conditions promote bacterial dormancy and metabolic quiescence, thereby conferring an intrinsic tolerance to antibiotic treatment. This multilayered defense mechanism renders biofilm‐associated infections (BAI) exceptionally stubborn and significantly complicates clinical treatment [9]. Among all BAI cases, chronic wound infections caused by methicillin‐resistant *Staphylococcus aureus* (MRSA) stand out as particularly prominent and challenging [10]. As a highly resistant Gram‐positive bacterium, MRSA not only exhibits resistance to multiple commonly used antibiotics but also readily forms robust biofilms in wound environments, posing a major bottleneck to wound healing [11, 12]. Consequently, the development of novel and efficient therapeutic strategies targeting MRSA biofilm infections has become an urgent imperative.

In clinical practice, to prevent the emergence of antibiotic‐resistant strains and effectively combat bacteria‐related infections associated with biofilms, it is essential to maintain sufficiently high antibiotic concentrations at the site of infection [13]. However, the formation of biofilms by resistant bacteria in wounds poses a significant challenge to traditional antibiotic treatment. Therefore, the development of antibiotic‐free strategies to address the increasingly severe problem of antibiotic resistance is urgently needed [14]. Glycosylated macrocyclic antibiotics, peptide‐modified macrocycles, and cationic macrocycle‐based antibacterial materials have emerged as promising therapeutic strategies [15, 16]. However, bacteria can develop resistance through various mechanisms, such as altering outer membrane permeability or activating efflux pumps to evade glycosylated macrocycles, producing specific peptide‐degrading enzymes, or modifying membrane surface charge density and outer membrane structure to diminish the efficacy of cationic macrocycles. These adaptive strategies can significantly undermine therapeutic effectiveness [17]. Antibacterial photodynamic therapy (APDT), as an emerging antibiotic‐free technology, demonstrates significant potential in the field of antimicrobial therapy by leveraging the unique mechanism of action involving the synergistic effects of light, oxygen, and photosensitizers (PS) to destroy microorganisms [18, 19]. The advantage of APDT lies in its ability, after drug penetration, to induce oxidative stress under light exposure, weakening the biofilm matrix strength, disrupting bacterial adhesion, and altering their metabolic activity [20, 21]. This therapy exerts its bactericidal or bacteriostatic effects through photosensitizer‐mediated molecular mechanisms [22, 23]. However, APDT faces inherent limitations, including poor stability, susceptibility to aggregation, limited water solubility, and occasional photobleaching, which can compromise the optimal efficacy of PS and photothermal agents. Additionally, short‐wavelength light exhibits weak penetration into biological tissues, and light‐responsive nanoparticles tend to detach from the target site, leading to a short release distance and limited lifespan of reactive oxygen species (ROS). Furthermore, the dense structure of biofilms restricts the penetration of both drugs and PS, while light attenuation within tissues further hinders the widespread clinical application of APDT [24, 25]. Recent advances have shown that visible light–activated antibacterial therapies enable site‐specific targeting of infections with high efficacy [26]. Therefore, a series of rationally designed synergistic strategies are needed to enhance the effectiveness and applicability of APDT. Peroxide‐based chemiluminescence is one of the most commonly used chemical luminescent substrates after luminol [27]. In the presence of hydrogen peroxide (H_2_O_2_), bis(2,4,5‐trichloro‐6‐carbopentoxyphenyl) oxalate (CPPO) undergoes oxidative decomposition, generating 2‐hydroxy‐3,5,6‐trichlorobenzene carboxylic acid pentyl ester along with a high‐energy intermediate, 1,2‐dioxetanedione [28, 29]. During this process, CO_2_ is concomitantly released [30]. The relaxation of this high‐energy intermediate produces chemiluminescence, which has been exploited in tumor photodynamic therapy (PDT) to activate porphyrin‐based PS and achieve effective antitumor responses [31]. During the peroxyoxalate chemiluminescence process, the decomposition of CPPO in the presence of H_2_O_2_ produces a high‐energy 1,2‐dioxetanedione intermediate, which subsequently transfers its energy to the photosensitizer via chemiluminescence resonance energy transfer (CRET). This nonradiative energy transfer promotes the photosensitizer from its ground‐state highest occupied molecular orbital (HOMO) to the excited lowest unoccupied molecular orbital (LUMO), analogous to conventional optical excitation. Compared to traditional photoexcitation, CPPO's chemical luminescent properties avoid light scattering and photodegradation effects, and its luminescence intensity is unaffected by oxygen concentration, thus offering unique advantages. It is noteworthy that MRSA produces H_2_O_2_ as a byproduct during metabolism [31, 32]. Moreover, wound tissues infected by MRSA undergo a series of redox imbalance reactions due to hypoxia and injury, further promoting local accumulation of H_2_O_2_. Especially in the early stages of infection, the H_2_O_2_ level at the wound site is relatively high, ranging from 20 to 100 μM, sufficient to trigger CPPO luminescence and CO_2_ gas production [33]. The dense architecture and adhesive EPS matrix of biofilms severely impede the penetration of exogenous agents. As a small gaseous molecule, CO_2_ can disrupt biofilm structural integrity and, by generating a transient “gas cushion,” facilitate nanocarrier propulsion and reduce penetration resistance [34]. Additionally, gas molecule diffusion is robust, enabling the formation of larger diffusion areas within the biofilm, expanding the action range of nanocarriers beyond the vicinity of the entry point to cover the entire biofilm.

An increasing body of research indicates that hydrogen sulfide (H_2_S) gas plays crucial roles as a cellular protector and redox regulator in the natural environments of prokaryotic organisms [35]. Analysis of bacterial genomes reveals that most bacteria possess homologous genes related to H_2_S production enzymes found in mammals, suggesting the evolutionary conservation of this metabolic pathway and its importance in bacterial cellular functions [36]. Key enzymes involved in H_2_S generation include cystathionine β‐synthase (CBS), 3‐mercaptopyruvate sulfur transferase (3MST), and cystathionine γ‐lyase (CSE) [37]. Staphylococcus aureus strains (MSSA RN4220 and MRSA MW2) harbor CBS/CSE operons but lack 3MST. Notably, MRSA infection often coincides with alterations in host and pathogen redox physiological states. Emerging studies reveal that bacterially derived H_2_S constitutes a defense system against antibiotics and oxidative stress [38, 39]. H_2_S‐producing enzymes counteract the bacteriostatic effects of antibiotics by generating H_2_S and balancing ROS levels within the organism [40]. Therefore, inhibiting bacterial H_2_S‐producing enzymes reduces bacterial H_2_S levels, disrupting this balance, leading to increased ROS levels within bacteria and exerting bactericidal effects [41]. Hypericin, an extract from Hypericum perforatum, belongs to the anthraquinone class of compounds and is one of its most biologically active components [42]. Clinically, hypericin is used to prevent and treat influenza viruses and exhibits antibacterial properties. Recent studies have found that Hypericin can inhibit CBS enzyme activity, thereby suppressing H_2_S production and disrupting bacterial resistance mechanisms [43]. Notably, hypericin functions as a photosensitizer to generate ROS upon irradiation, while in the dark it suppresses the antiapoptotic protein Bcl‐2 to sensitize tumor cells to death; independently, it also inhibits CBS enzyme activity, dismantling MRSA's H_2_S‐based self‐defense. This dual “photodynamic‐plus‐enzyme‐inhibition” modality thus potentiates both anticancer and antibacterial outcomes [44, 45]. When activated by light, hypericin generates singlet oxygen, which can accumulate excessively and damage biomolecules such as RNA, DNA, proteins, and lipids, triggering oxidative stress reactions [46]. Therefore, Hypericin not only possesses excellent anticancer and bactericidal abilities but also combats bacterial H_2_S defense systems, making it an outstanding candidate for APDT applications.

**FIGURE 1:**
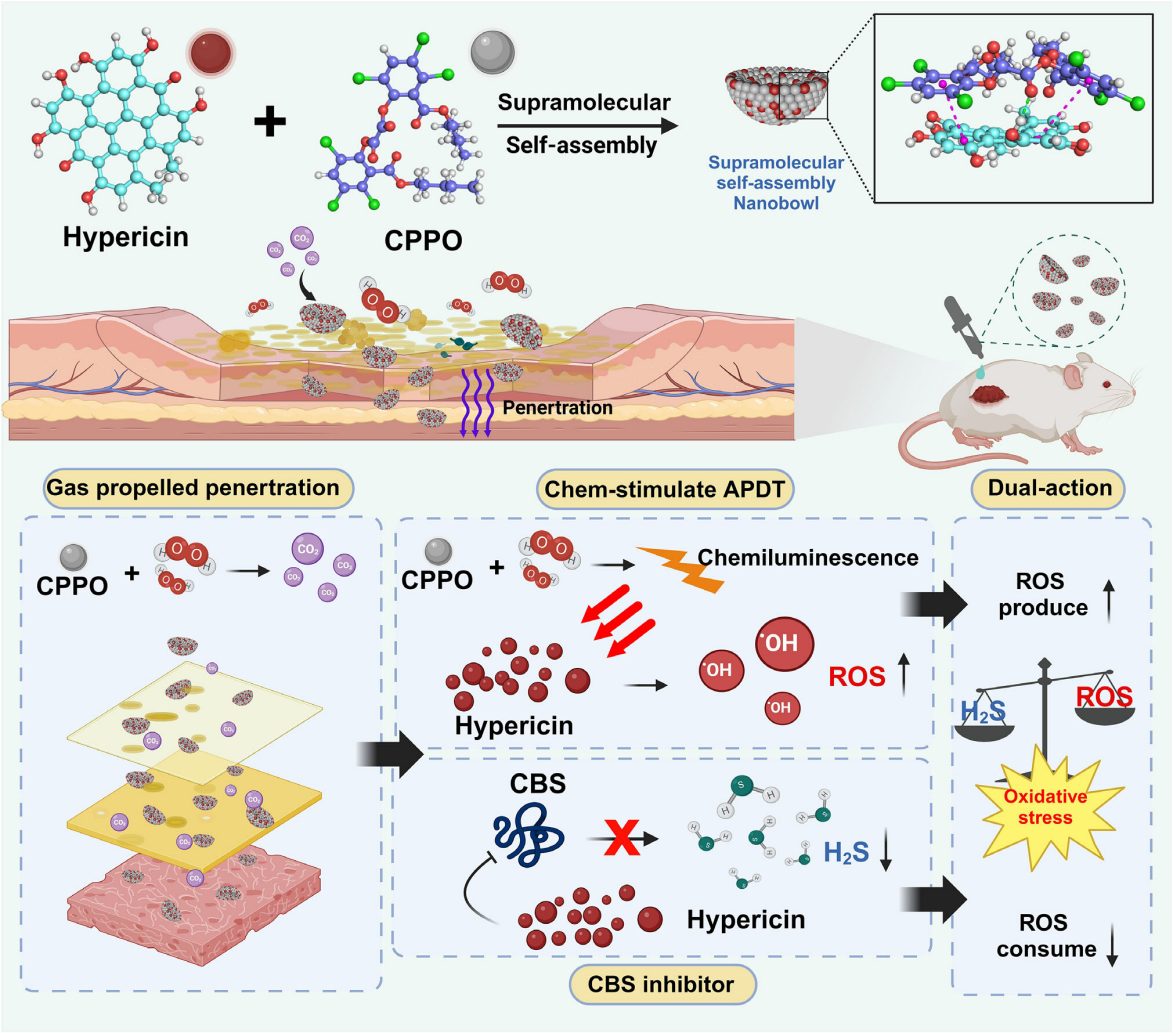
Schematic diagram for chem‐stimulated antibacterial photodynamic therapy system (SSA NBs). This system activates antibacterial photodynamic therapy through chemiluminescence while simultaneously generating CO_2_, which facilitates the deep penetration of SSA NBs into the biofilm. Additionally, the intrinsic CBS enzyme inhibition by hypericin successfully inhibits H_2_S production, overcoming the self‐protection mechanism of the bacterial biofilm.

Supramolecular self‐assembly refers to the process in which molecules spontaneously form ordered structures through noncovalent interactions such as hydrogen bonding, π–π stacking, hydrophobic interactions, and electrostatic attraction [47]. Self‐assembled structures can integrate PS, metal ions, and antimicrobial peptides, enabling multimodal antibacterial action through physical membrane disruption, chemical oxidative damage, and biological signal interference [48, 49]. In summary, we use a supramolecular self‐assembly strategy to integrate CPPO and hypericin, aiming to achieve chem‐stimulate APDT. We ultimately obtain nanoparticles with a bowl‐like structure, named as supramolecular self‐assembled nanobowls (SSA NBs). By exploiting the in situ chemiluminescence arising from the chemical reaction between H_2_O_2_ accumulated at MRSA‐infected wound sites and CPPO, this approach cleverly circumvents the limitations of limited light penetration depth and biofilm resistance encountered by traditional PDT, thereby significantly enhancing therapeutic efficacy. It is noteworthy that another product of the CPPO‐H_2_O_2_ reaction, dioxocyclobutanone, further decomposes to generate CO_2_, whose released gas provides propulsion for SSA NBs, facilitating deep penetration through biofilms and disruption of the dense biofilm structure formed by MRSA. Once hypericin is effectively stimulated by chemiluminescence, it generates ROS intrinsically, inducing oxidative stress and weakening the MRSA biofilm matrix structure. Furthermore, hypericin possesses inherent antibacterial activity and the ability to inhibit CBS, disrupting the self‐protective mechanism of MRSA against H_2_S oxidative stress, thereby enhancing the overall antibacterial efficacy of SSA NBs. This strategy harnesses the high concentration of H_2_O_2_ on the wound biofilm surface to activate PDT via in situ chemiluminescence, overcoming the limitation of weak external laser penetration. Additionally, the nanoparticles exert their effects directly at the infection site, addressing the issue of the short release distance of ROS. Skin infections are often accompanied by localized inflammation and bacterial aggregation. In situ chemiluminescence‐activated antibacterial PDT enables precise, site‐specific treatment at the infection site, effectively avoiding the antibiotic resistance associated with conventional therapies while enhancing therapeutic efficacy (refer to Figure 1). In summary, utilizing chem‐stimulated APDT for antibacterial therapy represents an innovative dual‐action strategy. This approach not only overcomes the limitations of light penetration depth but also enables precise activation of multiple antibacterial mechanisms, offering a novel solution for treating recalcitrant MRSA infections and associated diseases with promising clinical translational prospects.

## 2 Results and Discussion

### 2.1 Preparation and Characterization of SSA NBs

**FIGURE 2:**
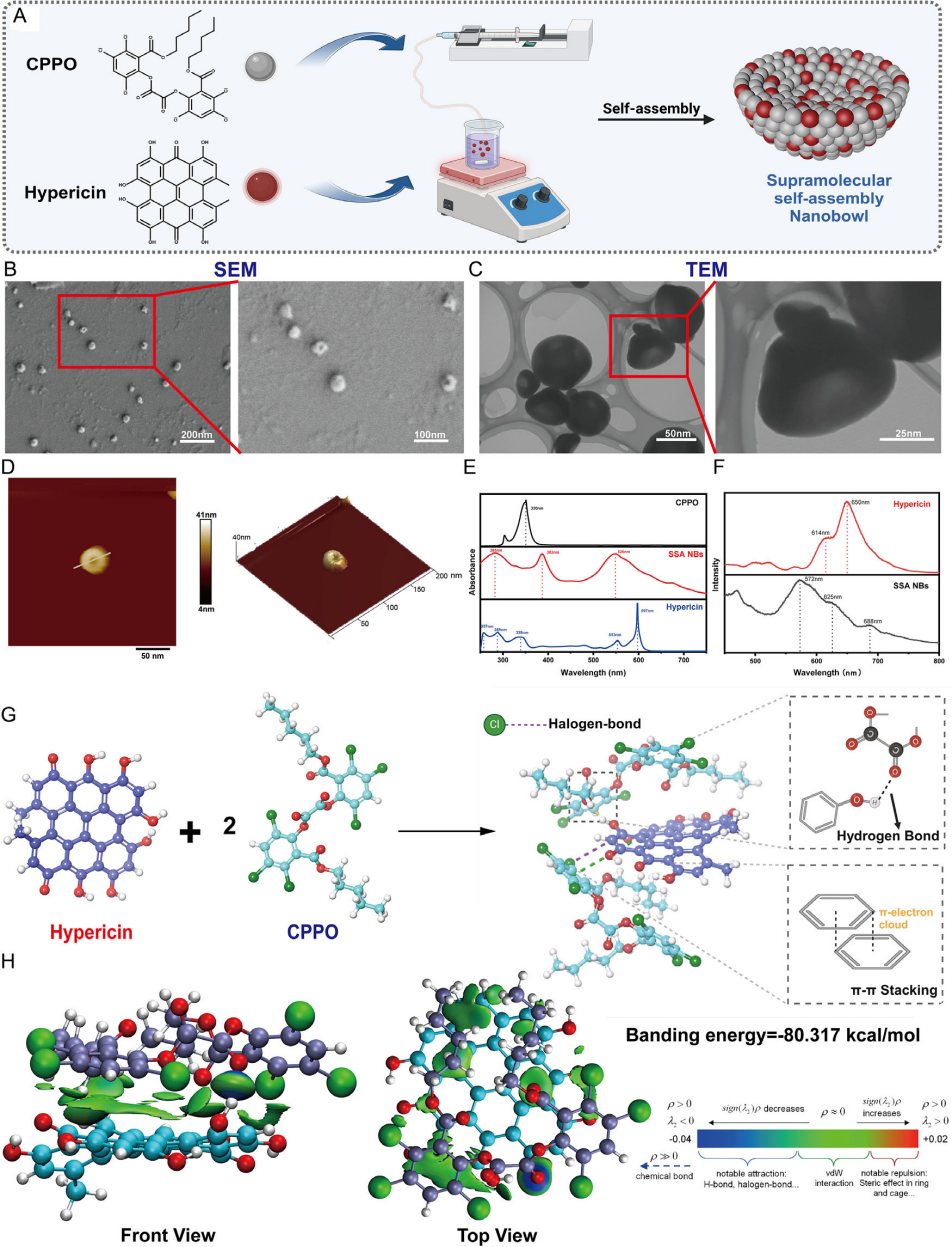
Preparation and characterization. (A) Schematic Illustration of SSA NBs Preparation; (B) SEM images and enlarged views of SSA NBs (Scale Bar: 200 nm); (C) TEM images and enlarged views of SSA NBs (Scale Bar: 200 nm); (D) particle size (nm) and PDI of SSA NBs; (E) UV absorption characteristic peak diagram of hypericin, CPPO, and SSA NBs; (F) fluorescence spectra of hypericin and SSA NBs; (G) molecular docking schematic diagram, binding forces, and binding energies of hypericin and CPPO; (H) schematic diagram of MIF at different angles. Data represent mean ± SD; *p < 0.05, **p < 0.01, ***p < 0.001, ****p < 0.0001. ns: not significant.

SSA refers to the spontaneous aggregation of organic molecules through noncovalent supramolecular forces (hydrophilic and hydrophobic, hydrogen bonds, electrostatic, host and guest, π–π stacking, and van der Waals forces) to form stable aggregates. In this work, CPPO and hypericin were integrated via an SSA strategy. A CPPO solution was injected into a hypericin solution at a controlled rate using an infusion pump, enabling spontaneous assembly in solution. This process yielded bowl‐shaped nanoparticles, termed SSA NBs (Figure 2A, S1). We observed the obtained nanoparticles through scanning electron microscopy (SEM) and transmission electron microscopy (TEM), revealing a distinct bowl‐shaped morphology with uniform size distribution around 50 nm (Figure 2B,C). Further characterization of the nanoparticles using atomic force microscopy (AFM) confirmed the distinct bowl‐like concavity of the SSA NBs (Figure 2D). The measured particle size was ≈50 nm, with an inward concavity depth of 15.3 nm. These findings, combined with previous characterization results, demonstrate the successful fabrication of SSA NBs with uniform size, unique morphology, and excellent dispersibility. To verify whether CPPO and hypericin molecules were successfully loaded into SSA NBs, we performed UV‐visible (UV–vis) absorption spectroscopy analysis (Figure 2E). In the UV spectrum, CPPO exhibits characteristic absorption peaks, with the B‐band representing the benzene ring absorption of aromatic compounds and a strong absorption peak at 330 nm corresponding to the K‐band, which results from π–π* transitions in conjugated double bonds. Similarly, in the UV spectrum of hypericin, absorption peaks at 257 and 289 nm are also attributed to the B‐band of benzene rings in aromatic compounds. A weaker absorption peak at 338 nm, identified as the R‐band, arises from n‐π* transitions associated with the absorption of heteroatomic unsaturated groups (C=O). The peaks at 553 and 597 nm correspond to the compound's charge‐transfer absorption bands, reflecting intramolecular charge‐transfer processes. Upon combination, a broad peak appeared at 285 nm, indicating a blue shift in the B‐band absorption of benzene rings due to an extension of the conjugated system or a change in electron cloud distribution. Additionally, the absorption peak near 300 nm underwent a redshift to 350 nm, as the formation of a larger conjugated system reduced the energy required for π–π* transitions, resulting in redshift. The disappearance of the charge‐transfer absorption band at 597 nm suggests that the interaction between CPPO and hypericin disrupted hypericin's original conjugated system, preventing electronic transitions that previously occurred at 597 nm. The absorption peak at 553 nm exhibited a blueshift to 536 nm, indicating the formation of a new, larger conjugated system. The expansion of electron delocalization in this new system altered energy levels, causing a blueshift in the absorption wavelength. To further examine this peak shift phenomenon, we conducted fluorescence spectroscopy (Figure 2F). In the fluorescence spectrum of hypericin, emission peaks at 614 and 650 nm correspond to transitions between different molecular energy levels, with the broad peak around 650 nm representing a typical fluorescence feature of hypericin. These peaks are associated with hypericin's excited‐state emissions, characteristic of anthraquinone derivatives. The fluorescence spectrum of SSA NBs displayed three distinct emission peaks at 572, 625, and 688 nm. Compared with free hypericin, these spectral changes indicate strong interactions between hypericin and CPPO, which modify the electronic environment of hypericin and give rise to multiple excited states. The additional emission peaks at 572 and 688 nm are likely associated with energy or charge‐transfer processes between hypericin and CPPO upon complex formation. Additionally, the fluorescence peak at 625 nm may be associated with the formation of novel electronic states during the assembly process. The π‐electron cloud of hypericin might overlap with the aromatic rings of CPPO, generating new excited states or excited‐state combinations, thereby altering fluorescence characteristics and manifesting as the fluorescence peak at 625 nm. The shift in fluorescence peak positions in SSA NBs reflects the significant impact of intermolecular interactions on their optical properties. Additionally, the FTIR spectrum (Figure S2) of SSA‐NBs simultaneously preserves all characteristic skeletal vibrational bands of hypericin and CPPO (1600, 1570, 845, and 760 cm^−1^, etc.), while four bands at 3400, 1765, 1725, and 1680 cm^−1^ exhibit redshifts of ≈10–15 cm^−1^. These spectral shifts indicate the formation of OH and O=C hydrogen bonds between the phenolic hydroxyl groups of hypericin and the ester carbonyl groups of CPPO, while the C—Cl moieties of CPPO participate in halogen bonding, collectively driving the SSA. To further quantify the drug loading capacity of CPPO in SSA NBs, we employed high‐performance liquid chromatography (HPLC) analysis and established a standard curve for CPPO at different concentrations based on its specific retention time. For hypericin, a standard curve was constructed based on its characteristic UV absorption peak at 592 nm (Figure S3). By measuring the peak area at a retention time of 5 min and 43 s at a wavelength of 235 nm, the results indicated that the CPPO content in SSA NBs was 0.87 mg per 1 mg of SSA NBs. Meanwhile, UV–vis spectroscopy determined the hypericin content to be 0.103 mg. To further explore the interaction mechanism between CPPO and hypericin within SSA NBs, we conducted molecular docking simulations. The results demonstrated that CPPO molecules could firmly bind to hypericin molecules. As shown in Figure 2G, two CPPO molecules align parallel to the hypericin molecule, forming a stable complex primarily through hydrogen bonding, halogen bonding, and π–π stacking interactions between aromatic rings. The calculated binding free energy was −80.317 kcal/mol, indicating the formation of a relatively stable complex. Furthermore, molecular interaction field (MIF) calculations of molecular interaction strength (Figure 2H) further confirmed the presence of strong hydrogen bonding interactions (represented by blue isosurfaces), van der Waals forces (green isosurfaces), and significant π–π stacking interactions between CPPO and hypericin, further verifying their stable binding. Previous studies indicate that the self‐assembly of these components is governed by synergistic π–π stacking, hydrophobic interactions, and hydrogen bonding. By adjusting the precursor ratios, the opening and inner pore sizes of the NBs can be precisely tuned, suggesting that these noncovalent interactions between hypericin and CPPO drive the formation of the bowl‐like architecture.

### 2.2 Evaluation of the Self‐Activation Process and Movement Trajectory of SSA NBs

**FIGURE 3:**
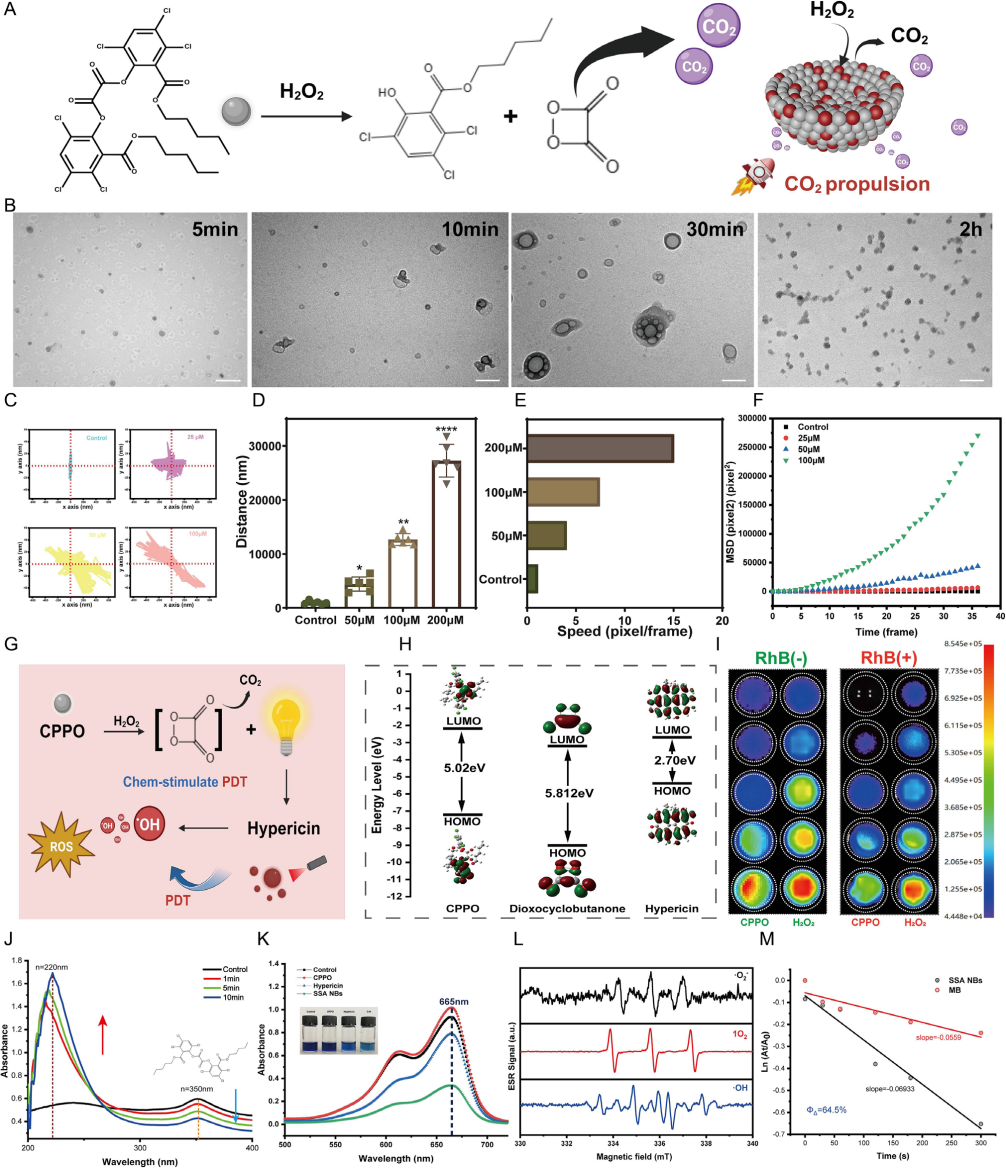
Study on H_2_O_2_‐activated APDT. (A) Schematic diagram of CPPO reacting with H_2_O_2_ to produce CO_2_ as propulsion for SSA NBs; (B) TEM images of SSA NBs at different time points upon exposure to H_2_O_2_ (Scale Bar: 200 nm); (C) trajectories of SSA NBs under different concentrations of H_2_O_2_; (D) movement distance (nm) of SSA NBs under different concentrations (*n* = 5); (E) average velocity of SSA NBs under different concentrations of H_2_O_2_ (1 pixel represents the size of a nanoparticle, 30 frames = 1s); (F) mean square displacement (MSD) of SSA NBs under different concentrations of H_2_O_2_; (G) schematic diagram of chem‐stimulated APDT mechanism; (H) HOMO/LUMO energy level orbitals of tetrahydroquinoline and hypericin in the activation mechanism; (I) fluorescence imaging of different concentrations of hydrogen peroxide and CPPO, and fluorescence imaging with the dye rhodamine (562 nm); (J) changes in absorption peaks of CPPO in SSA NBs before and after activation, and enhancement of absorption peak at 220 nm; (K) UV absorption spectra by MB colorimetric method of control group, CPPO, hypericin, and SSA NBs after interaction; (L) ESR spectra of ROS generated by SSA NBs; (M) quantum yield of singlet oxygen produced by SSA NBs. Each data bar represents mean ± SD with 5 technical replicates unless otherwise indicated; *p < 0.05, **p < 0.01, ***p < 0.001, ****p < 0.0001. ns: not significant. One‐way ANOVA test, Dunnett's multiple comparisons test in comparison to the control.

In the SSA NBs system, CPPO reacts with wound‐site H_2_O_2_ to generate CO_2_, producing propulsion that promotes deep penetration into MRSA biofilms. To verify this effect, morphological changes of SSA NBs in the presence of H_2_O_2_ were examined by TEM (Figure 3A). Compared to the control group, SSA NBs exhibited signs of morphological changes at 10 min after the addition of H_2_O_2_, with a significant increase in particle size and the appearance of internal pore structures at 15 min (Figure 3B). This phenomenon can be attributed to the reaction between CPPO and H_2_O_2_, resulting in the production of CO_2_ gas. Importantly, when the reaction time was extended to 30 min, the size of SSA NBs significantly decreased, suggesting the occurrence of some degree of degradation. To further investigate the effect of gas production on the mobility of SSA NBs, we employed particle tracking analysis to evaluate the dynamic parameters such as motion trajectories, displacement distance, and velocity of SSA NBs under different concentrations of H_2_O_2_. As shown in Figure 3C and D, compared to the control group, the displacement distance of SSA NBs increased significantly with the elevation of H_2_O_2_ concentration at the same time interval. Further analysis of the motion velocity of SSA NBs (Figure 3E) revealed that at an H_2_O_2_ concentration of 20 μM, the average motion velocity of SSA NBs (15.074 pixels/frame) was nearly 15 times higher than the Brownian motion of the control group (1.12 pixels/frame). Additionally, we calculated the mean squared displacement (MSD) of SSA NBs (Figure 3F) to assess their long‐distance mobility. The MSD value reflects the average displacement distance of micro/nanomotors relative to their initial position, depending on both motion velocity and directionality. The MSD reflects the overall mobility of nanomotors by integrating motion velocity and directionality. As the H_2_O_2_ concentration increased, the MSD of SSA NBs rose markedly, consistent with velocity analyses, indicating that H_2_O_2_‐triggered gas generation enhances both propulsion efficiency and directional motion. As shown in Figures S4–S7, higher H_2_O_2_ levels led to more vigorous movement, longer travel distances, and faster speeds. This enhanced motility enables SSA NBs to overcome the physical barriers of MRSA biofilms, facilitating deep biofilm penetration and supporting subsequent self‐activated PDT.

### 2.3 Analysis of the Self‐Activation Process of SSA NBs in Exerting APDT

In traditional PDT, PS such as hypericin require specific wavelengths of external light sources to be excited to generate ROS for antibacterial action. In this study, we propose an innovative self‐activation strategy using the chemiluminescence produced by the reaction between CPPO and H_2_O_2_ to replace external light sources, thereby intrinsically activating hypericin to exert photodynamic antibacterial efficacy (Figure 3G). To achieve the chemical excitation of hypericin by CPPO, there must be a certain energy gap between them to ensure electron transfer between the molecules. Using density functional theory (DFT), we calculated the HOMO energy level of hypericin (−5.38 eV) and the LUMO energy level of CPPO (−2.18 eV), revealing an energy gap of ≈3.2 eV (Figure 3H). In addition, the photodynamic activation of hypericin by CPPO is achieved through a cascade chemiluminescence reaction, the thermodynamic feasibility of which is determined by the total Gibbs free energy (ΣΔG). Specifically, the first step—the reaction between CPPO and methylpyridine (H_2_O_2_)—is spontaneous (ΔG_1_ < 0), generating the high‐energy intermediate 1,2‐dioxetanedione. When this intermediate transitions from the ground state to the excited state, it releases ≈5.812 eV of energy, which is sufficient to excite hypericin molecules. This result indicates the inherent spontaneity of the process (Figure S8). To further confirm the activation and luminescence process, fluorescence assays were performed using varying concentrations of hydrogen peroxide and CPPO. The results showed a concentration‐dependent increase in fluorescence intensity. Consistently, colorimetric experiments using rhodamine (emission at 562 nm) exhibited a similar enhancement in fluorescence with increasing hydrogen peroxide and CPPO levels (Figure 3I), indicating that the reaction produces light energy, which can be absorbed by the fluorescent dye, supporting subsequent APDT. Considering the chemical structural changes of CPPO during activation, we characterized it using UV–vis spectroscopy (Figure 3J). The results showed that with prolonged reaction time, the characteristic absorption peak of CPPO in the SSA NBs solution (350 nm) gradually weakened, while a new absorption peak appeared at 220 nm, with its intensity showing a negative correlation with the CPPO absorption peak. Based on theoretical calculations, we hypothesized that this new absorption peak likely corresponds to the product formed after CPPO undergoes deprotonation and the removal of two CO_2_ molecules, resulting in 2‐hydroxy‐3,5,6‐trichlorobenzene pentanoate. Notably, bacterial infections in wound areas are often accompanied by the accumulation of H_2_O_2_, providing an ideal chemical excitation source for self‐activated APDT. Subsequently, we used a methylene blue (MB) probe experiment to assess the levels of ROS production under different conditions. Compared to the control group, the MB absorption peak at 652 nm for SSA NBs decreased by approximately threefold (Figure 3K), indicating high ROS generation efficiency. This phenomenon is likely due to the enhanced chemiluminescence of CPPO at higher H_2_O_2_ concentrations, which in turn induces hypericin to generate more ROS. To further investigate the types of ROS produced, we conducted electron spin resonance (ESR) analysis. The results (Figure 3L) indicated the presence of superoxide anions, hydroxyl radicals, and singlet oxygen, with singlet oxygen being the dominant species. Subsequently, we quantified the singlet oxygen quantum yield using MB and DPBF detection. The results (Figure 3M) showed that the quantum yield (*Φ*) was 64.5%. Collectively, these findings confirm the unique advantage of SSA NBs in self‐activated photodynamic antibacterial therapy.

### 2.4 Analysis of SSA NBs Inhibiting the Production of H_2_S by MRSA

**FIGURE 4:**
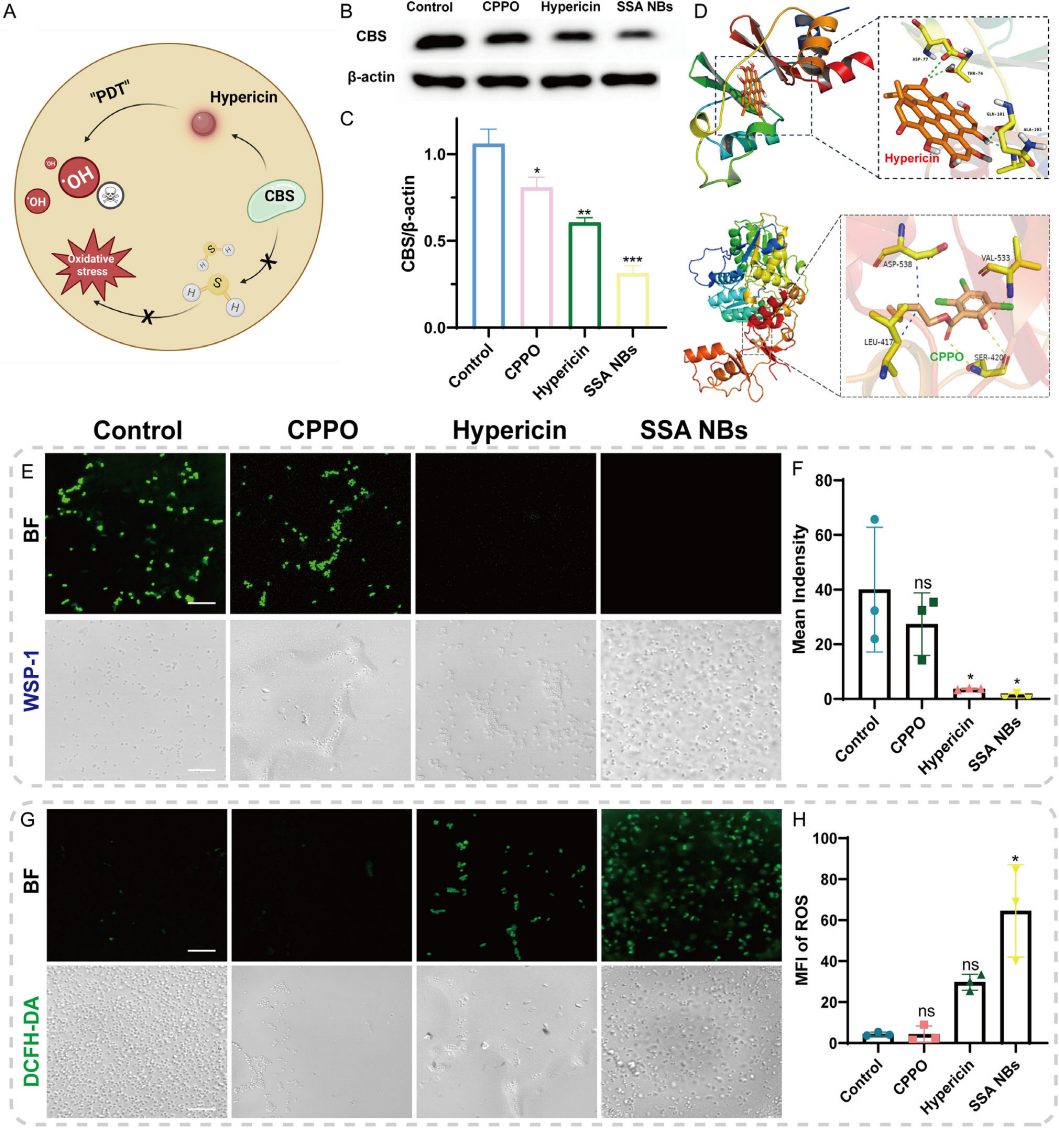
Study on the inhibition of bacterial H_2_S by SSA NBs. (A) MRSA's endogenous H_2_S defense mechanism and the mechanism of action of APDT; (B) Western blot of CBS enzyme in MRSA after treatment with different groups (*n* = 3); (C) quantitative analysis of WB results by ImageJ (*n* = 3); (D) molecular docking simulation of CPPO and 2‐hydroxy‐3,5,6‐trichlorobenzoyl pentanoate with CBS enzyme; (E) levels of H_2_S in MRSA bacteria after administration in different groups (Scale Bar: 50 μm) (*n* = 3); (F) quantification of WSP‐1 green fluorescence for H_2_S (*n* = 3); (G) CLSM fluorescence images of ROS production after administration in different groups (Scale Bar: 50 μm) (*n* = 3); (H) quantification of DCFH‐DA green fluorescence for ROS (*n* = 3). Each data bar represents mean ± SD with 3 technical replicates unless otherwise indicated; *p < 0.05, **p < 0.01, ***p < 0.001, ****p < 0.0001. ns: not significant. One‐way ANOVA test, Dunnett's multiple comparisons test in comparison to the control.

MRSA can form an endogenous self‐protection mechanism by producing H_2_S, which helps to resist host immune damage and antibiotic attacks. We hypothesize that the inhibition of cystathionine β‐synthase (CBS) activity by hypericin and the generation of ROS from chem‐stimulated PDT may synergistically disrupt this protective mechanism of MRSA, thereby cutting off the source of H_2_S production from a dual perspective (Figure 4A). First, we examined the effect of different treatments on the expression levels of CBS protein in MRSA using Western blotting (Figure 4B,C). Results demonstrated a significant reduction in CBS expression with hypericin treatment alone, achieving an inhibition rate of 86%. Remarkably, in the SSA NBs group, CBS inhibition escalated to 98%, possibly attributed to ROS involvement in CBS expression suppression during the photodynamic process. Intriguingly, CPPO treatment alone also notably downregulated CBS expression, suggesting inherent or activated products may possess enzymatic inhibitory activity. To elucidate this phenomenon's molecular mechanism, molecular docking simulations were conducted to calculate the binding free energies of CPPO and its intermediate product, 2‐hydroxy‐3,5,6‐trichlorobenzyl pentanoate, with CBS enzyme. Results revealed a relatively weak binding affinity of CPPO (−5.2 kcal/mol), while 2‐hydroxy‐3,5,6‐trichlorobenzyl pentanoate exhibited stronger affinity (−6.5 kcal/mol), potentially exerting enzyme inhibitory effects through tight molecular bonding with key amino acid residues such as ASP‐538 and SER‐420 on the CBS enzyme. Hypericin bound to CBS enzyme through its carbonyl and ‐OH groups, forming strong interactions (−10.7 kcal/mol) with amino acid residues such as ASP‐77, THR‐74, GLH‐101, and ALA‐103 (Figure S9). Subsequently, we assessed the impact of different treatments on MRSA's H_2_S production capability using specific H_2_S fluorescent probes (Figure 4E). Compared to the control group, both the hypericin and SSA NBs treatment groups exhibited significantly reduced WSP‐1 signal values, further confirming their inhibitory effects on CBS enzyme activity and blockade of H_2_S synthesis. Finally, we measured the ROS levels in MRSA after various treatments (Figure 4F). The control group showed minimal ROS fluorescence (Figure 4G,H), while the CPPO and hypericin groups displayed faint fluorescence, possibly due to partial oxidative stress response resulting from reduced hydrogen sulfide production upon inhibition of the CBS enzyme by activated intermediate products of H_2_O_2_. Interestingly, after treatment with SSA NBs, bacterial swelling and rupture were observed under microscopy, accompanied by a significant ROS burst.

### 2.5 In Vitro Antibacterial Efficacy

**FIGURE 5:**
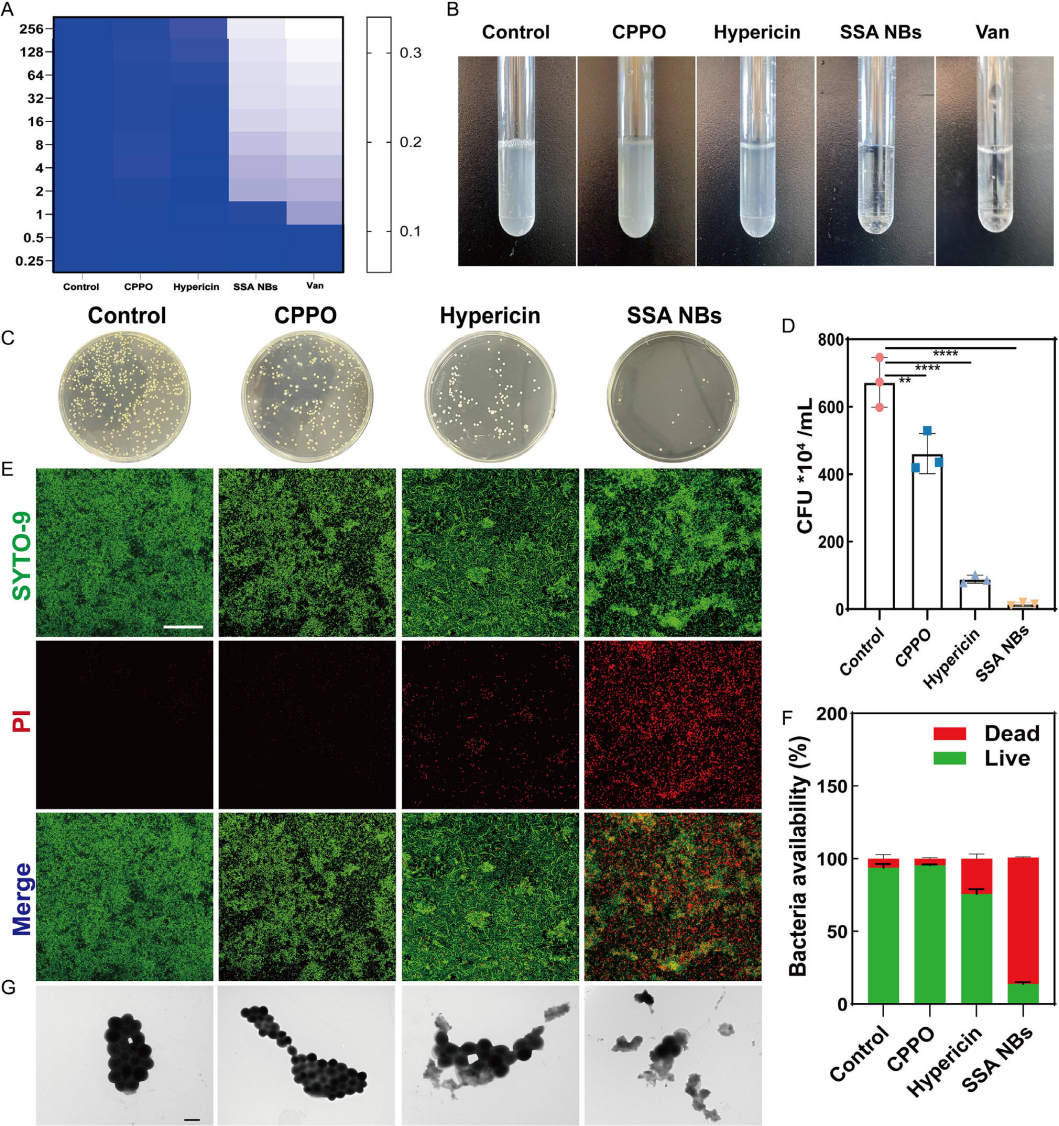
Analysis of the inhibitory effect of SSA NBs on bacterial suspensions. (A) Minimum inhibitory concentration (MIC) of CPPO, hypericin, SSA NBs, and positive control vancomycin determined by broth microdilution method (*n* = 3); (B) number of planktonic bacteria after cultivation in each group (PBS dispersal); (C) bacterial killing plate assay after administration of different formulations (*n* = 3); (D) statistical analysis of bacterial colony counts on TSB agar plates (*n* = 3); (E) viability of planktonic MRSA bacteria (SYTO‐9 green fluorescence staining for live bacteria, PI red fluorescence staining for dead MRSA bacteria) analyzed by CLSM imaging (Scale Bar: 50 μm) (*n* = 3); (F) analysis of viability of MRSA bacteria through fluorescence quantification; (G) morphological analysis of MRSA after treatment in different groups under TEM (Scale Bar: 5 μm). Each data bar represents mean ± SD with 3 technical replicates unless otherwise indicated; *p < 0.05, **p < 0.01, ***p < 0.001, ****p < 0.0001. ns: not significant. One‐way ANOVA test, Dunnett's multiple comparisons test in comparison to the control.

To comprehensively evaluate the antibacterial performance of SSA NBs, *Escherichia coli* and *Pseudomonas aeruginosa* were selected as representative Gram‐negative model strains, while MRSA was used as the typical Gram‐positive model. As shown in Figure S10, CPPO, hypericin, and SSA NBs exhibited no obvious inhibitory effects against the Gram‐negative strains. According to previous studies, the outer membrane of Gram‐negative bacteria is enriched with negatively charged lipopolysaccharides (LPS) formed by phosphate groups, which leads to electrostatic repulsion between ROS and the bacterial surface, thereby reducing binding efficiency. The outer LPS layer of Gram‐negative bacteria severely limits PS penetration, whereas Gram‐positive bacteria lack this barrier and possess a porous peptidoglycan structure that facilitates uptake. In addition, the relatively positive surface charge of Gram‐positive bacteria favors interactions with anionic PS. Because SSA NBs primarily generate negatively charged singlet oxygen, this explains their limited activity against *E. coli* and *P. aeruginosa* but enhanced antibacterial efficacy against Gram‐positive MRSA. Vancomycin (Van) was therefore selected as a positive control to evaluate the antibacterial activity of SSA NBs against planktonic MRSA. The minimum inhibitory concentration (MIC) of SSA NBs against MRSA was determined to be 2 μg/mL using the broth microdilution method (Figure 5A), indicating effective inhibition of MRSA growth at this concentration. Furthermore, we assessed the bactericidal effect of each group by coculturing bacteria with different formulations (Figure 5B). Compared to the control group, the number of surviving MRSA colonies in the SSA NBs‐treated group was significantly reduced (Figure 5C,D), with a quantitative analysis showing a killing rate of up to 98%, much better than the bactericidal levels achieved by using CPPO alone or hypericin alone. This finding was further supported by fluorescence viability/dead staining assays (Figure 5E,F). We observed that in the control group and the CPPO‐alone group, the majority of MRSA cells exhibited characteristic green fluorescence indicative of live bacteria, while in the hypericin‐alone group, the proportion of dead bacteria (red fluorescence) was slightly elevated (27.93%). In contrast, in the SSA NBs‐treated group, almost all MRSA cells were dead, with a survival rate of only 13.66%. These results collectively confirm that the SSA NBs nanosystem, through the synergistic effect of self‐activated photodynamic action with CPPO and the CBS enzyme inhibitory action of hypericin, exhibits remarkable anti‐MRSA activity. To elucidate the mechanism of action of SSA NBs against MRSA cells, we characterized the morphological changes of bacteria in different treatment groups using TEM (Figure 5G). MRSA cells in the control group and the CPPO‐alone group maintained typical spherical morphology with intact bacteria walls. In the hypericin‐alone group, some MRSA showed signs of swelling and deformation. Notably, after treatment with SSA NBs, MRSA exhibited the most severe damage, with ruptured bacteria walls and extensive leakage of cellular contents, indicating significant subcellular‐level damage. This observation further confirms the direct killing and destructive effects of SSA NBs on MRSA cells.

### 2.6 In Vitro Deep Biofilm Penetration and Antibiofilm Activity of and Viability of SSA NBs

**FIGURE 6:**
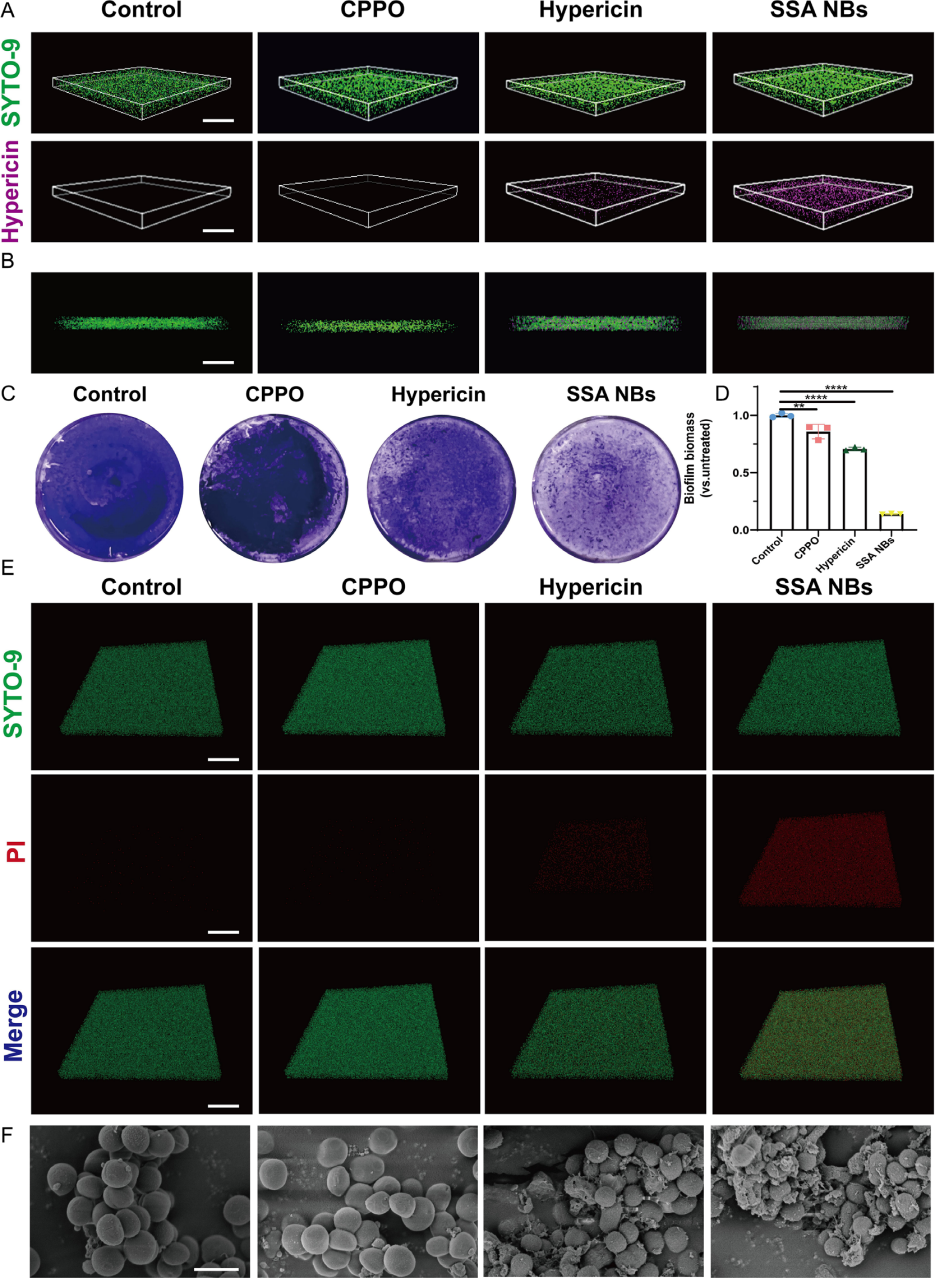
Analysis of the biofilm penetration and pharmacodynamics of SSA NBs. (A) Penetration layered CLSM images of MRSA biofilm treated with different formulations (scale bar: 100 μm); (B) merge images of formulation penetration (scale bar: 100 μm); (C) crystal violet staining method was used to evaluate the antibiofilm activity of samples against MRSA biofilms; (D) quantitative analysis of biofilm using crystal violet staining at an absorbance of 590 nm; (E) live/dead staining images of MRSA biofilms captured through 3D confocal laser scanning microscopy (scale bar: 100 μm); (F) SEM images of MRSA biofilms treated with different groups (scale bar: 2 μm). Each data bar represents mean ± SD with 3 technical replicates unless otherwise indicated; *p < 0.05, **p < 0.01, ***p < 0.001, ****p < 0.0001. ns: not significant. One‐way ANOVA test, Dunnett's multiple comparisons test in comparison to the control.

BFs refer to highly ordered three‐dimensional structures formed by bacterial communities enveloped in their self‐secreted EPS [50]. The polysaccharide‐rich biofilm matrix forms a physical barrier that limits antimicrobial penetration and efficacy. To overcome this challenge, we developed an SSA NBs nano‐antibiofilm system in which CPPO reacts with wound‐accumulated H_2_O_2_ to generate CO_2_, providing propulsion for deep biofilm penetration. Simultaneously, CPPO‐induced chemiluminescence activates encapsulated hypericin to trigger photodynamic antibacterial effects, enabling a dual assault on MRSA biofilms. Biofilm penetration was assessed using SYTO‐9–labeled biofilms and hypericin fluorescence (592 nm excitation) as a tracer. Compared with free hypericin, SSA NBs showed markedly enhanced penetration into MRSA biofilms (∼15 μm thickness) (Figure 6A,B), which may be attributed to the promoting effect of CO_2_ gas generated by CPPO on the penetration process. We further evaluated the antibacterial effect of SSA NBs on MRSA biofilms using crystal violet staining and viability/dead staining assays. As shown in Figure 6C, the crystal violet staining intensity of the biofilm after SSA NBs treatment was significantly reduced compared to other control groups, and quantitative analysis (Figure 6D) further confirmed its excellent biofilm removal ability. The results of viability/dead staining (Figure 6E) also clearly showed that almost all cells in the SSA NBs‐treated group exhibited red fluorescence of dead cells, indicating the complete destruction of the entire biofilm structure. SEM image analysis intuitively demonstrated the effects of different treatments on the microstructure of MRSA biofilms (Figure 6F). In the PBS control group, the biofilm maintained a dense and orderly reticular structure, with intact bacterial cell morphology. After hypericin treatment, although the overall structure of the biofilm was not severely damaged, some cells experienced membrane rupture. Of note, in the SSA NBs treatment group, formulation particles were present, leading to the complete disintegration of the biofilm structure, extensive rupture and dissolution of bacterial cells, and widespread detachment of the EPS matrix, resulting in the complete destruction of the entire biofilm structure, thereby rendering it incapable of maintaining its original functionality. These results demonstrate that the SSA NBs nanosystem exhibits excellent penetration capability and photodynamic antibacterial activity, exerting significant therapeutic efficacy against MRSA biofilms.

### 2.7 In Vivo Assessment of Antibiofilm Efficacy and Wound Healing Properties

**FIGURE 7:**
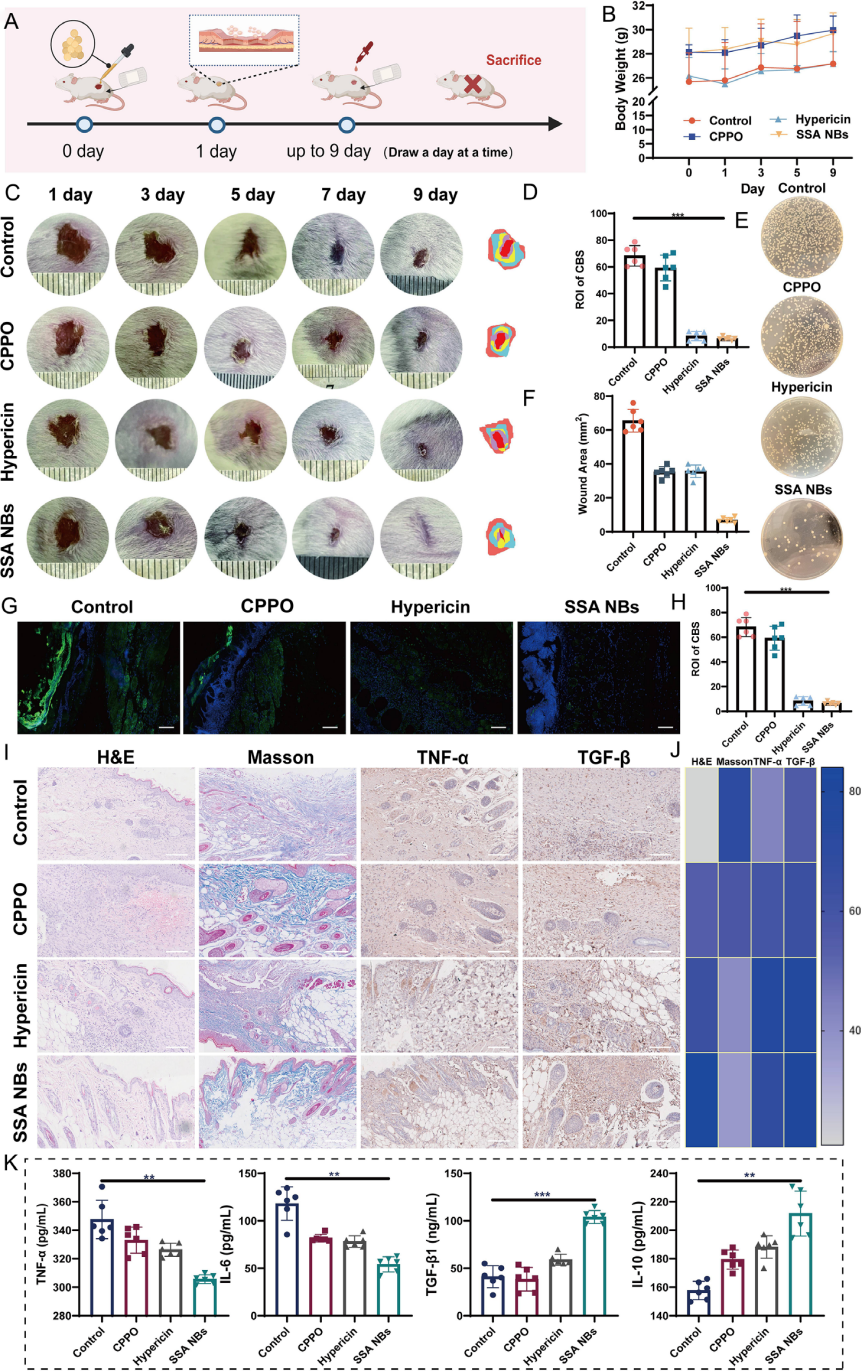
Pharmacodynamic study of SSA NBs in Vivo. (A) Schematic diagram of animal model construction and administration; (B) changes in body weight of mice after administration of different formulations (*n* = 6 per group); (C) visual condition of wounds on days 1, 3, 5, 7, and 9 after administration; (D) statistical analysis of wound area in mice after administration of different formulations; (E) wound swabbing results after treatment with different formulations (*n* = 6 per group); (F) bacterial count from wound swabs (*n* = 6 per group); (G) the fluorescent image of CBS staining on wound sections (Scale Bar: 100 μm); (H) the fluorescence quantification chart of CBS‐stained images (*n* = 6 per group). (I) H&E staining, Masson staining, and TNF‐α/TGF‐β Immunohistochemistry of wounds after treatment with different formulations (Scale Bar: 100 μm); (J) quantitative heat map of neutrophils in H&E staining, collagen fiber deposition in Masson staining, and TNF‐α/TGF‐β Immunohistochemistry using ImageJ; (K) statistical results of relevant inflammatory factors/growth factors (TNF‐α, IL‐6, IL‐10, TGF‐β) in mouse blood after treatment (*n* = 6 per group). Each data bar represents mean ± SD with 6 technical replicates unless otherwise indicated; *p < 0.05, **p < 0.01, ***p < 0.001, ****p < 0.0001. ns: not significant. One‐way ANOVA test, Dunnett's multiple comparisons test in comparison to the control.

The skin of the mice was excised, and then 200 μL of MRSA suspension (10^8^ CFU/mL) was added twice to form a wound with a diameter of 7 mm. After 24 h, the infection induced abscess formation, confirming successful wound establishment. The treatments, including PBS, CPPO, hypericin, and SSA NBs, were administered every other day, as shown in Figure 7A. Post‐treatment, there were no significant changes in body weight among the different groups, indicating good safety of the formulations (Figure 7B). The wound healing process was monitored, and wound areas were measured every other day. By day 9, wounds in the SSA NBs treatment group displayed scar formation and were nearly completely healed, whereas the control, CPPO, and hypericin groups still exhibited significant wounds (Figure 7C,D). Bacterial plating experiments on the wounds (Figure 7E,F) revealed minimal bacterial colonies in the SSA NBs‐treated wounds, unlike the other three groups, which showed substantial bacterial growth. Since SSA NBs combat the biofilm's self‐protection mechanism by inhibiting the H_2_S‐producing enzyme CBS in MRSA, we evaluated the in vivo efficacy of SSA NBs by assessing the fluorescence of CBS enzyme in the biofilm on the wound surface using fluorescence staining. As shown in Figure 7G and the quantitative analysis of CBS fluorescence in Figure 7H, the control group exhibited strong green CBS fluorescence, while the CPPO group did not show a significant decrease in fluorescence. In contrast, both the SSA NBs group and the hypericin group demonstrated a marked reduction in green fluorescence due to hypericin's inhibition of CBS. Additionally, the blue fluorescence represents DAPI‐stained cell nuclei, and the images reveal that after treatment with SSA NBs, there is significant fibroblast accumulation on the wound surface, which is notably more pronounced compared to the hypericin group. We further examined neutrophil infiltration, collagen deposition, and expression of related inflammatory factors in wound tissues (Figure 7I,J). H&E staining was used to assess tissue recovery in the infected wound sites. Wound healing restores skin integrity through the formation of granulation tissue and new epithelial tissue, which facilitates wound closure. Granulation tissue, characterized by proliferating capillaries, fibrous connective tissue, and various inflammatory cells, appears bright red and can fill wound gaps. In the early stages of wound healing, neutrophil infiltration is critical [51]. H&E staining results after 9 days of treatment showed that the SSA NBs treatment group had the most granulation tissue and highest degree of skin recovery, indicating enhanced tissue remodeling and regeneration. Neutrophil infiltration was significantly reduced in the SSA NBs treatment group, demonstrating effective mitigation of wound inflammation. Masson's trichrome staining was used to detect collagen deposition in the wound, with collagen fibers appearing blue and distinctly separated from other tissue components. As the wound heals, collagen deposition gradually increases, aiding in the maturation of granulation tissue into mature connective tissue. The results showed that, compared to the control group, the CPPO group, and the hypericin group, the SSA NBs treatment group had the highest content of blue‐stained collagen fibers in the Masson's trichrome sections. Immunohistochemical staining for pro‐inflammatory cytokines (tumor necrosis factor‐alpha (TNF‐α)) and transforming growth factor (TGF‐β) was performed to assess inflammation at the wound sites. In the SSA NBs treatment group, TNF‐α was evenly distributed, and TGF‐β concentration was favorable for wound healing, suggesting extensive wound closure. Additionally, we used ELISA to examine the expression of inflammation‐related cytokines (H&E staining, Masson staining, and TNF‐α/TGF‐β immunohistochemistry) in the blood of treated mice (Figure 7K). The SSA NB treatment group showed decreased expression of pro‐inflammatory cytokines, indicating reduced systemic inflammation. Elevated levels of the anti‐inflammatory cytokine interleukin (IL)‐10 and the growth factor TGF‐β1 suggest that the mice in the SSA NBs treatment group had progressed into the latter stages of wound healing.

### 2.8 Biosafety Study of SSA NBs

**FIGURE 8:**
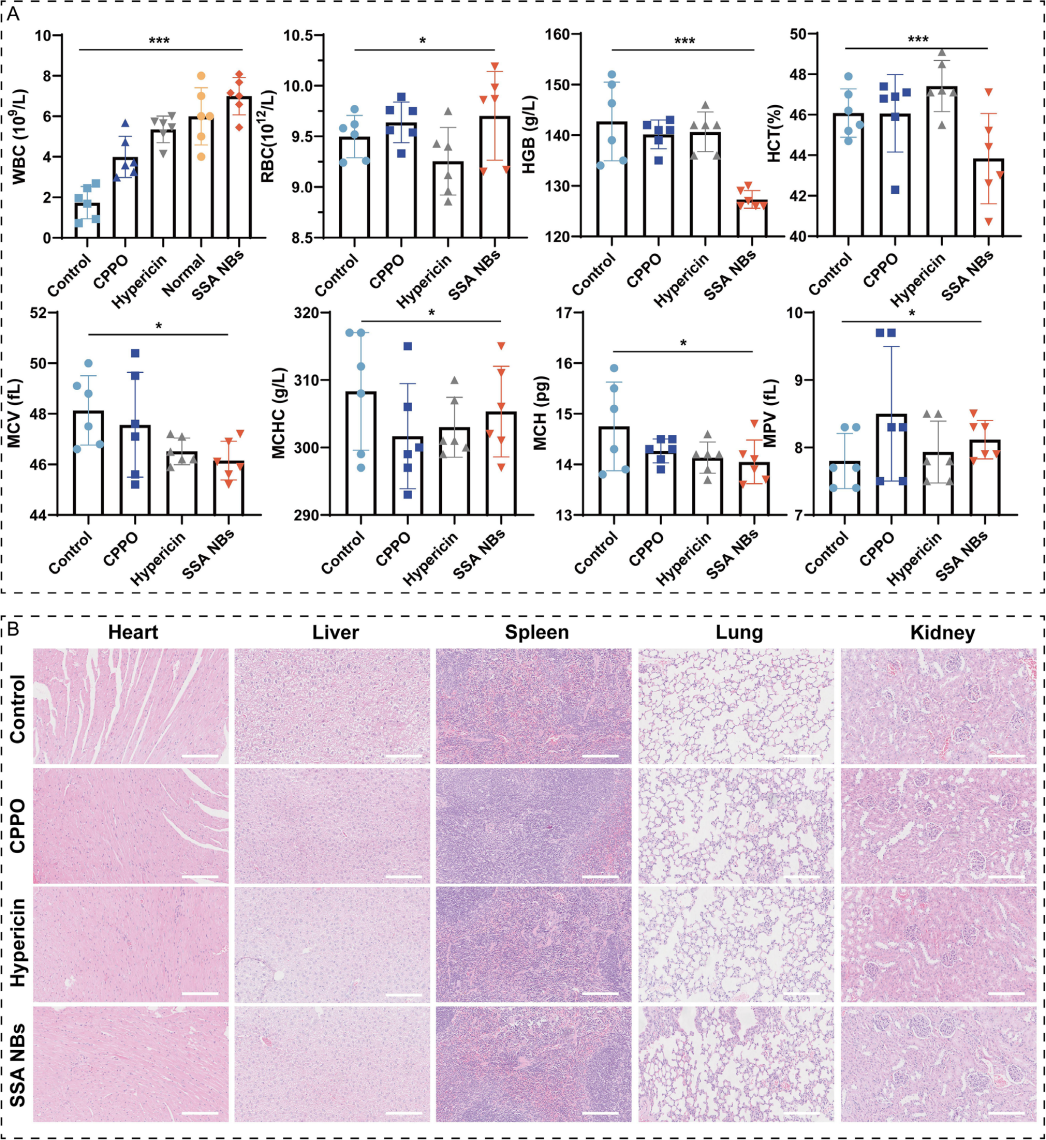
In vivo safety evaluation of SSA NBs. (A) Comparison of white blood cell (WBC) counts after treatment with different formulations to normal mice; statistical analysis of corresponding indicators (RBC, HGB, HCT, MCV, MCH, MCHC, MPV) after treatment with different formulations (*n* = 6 per group); (B) H&E staining of heart, liver, spleen, lung, and kidney sections after treatment with different formulations (Scale bar: 100 μm). Each data bar represents mean ± SD with 6 technical replicates unless otherwise indicated; *p < 0.05, **p < 0.01, ***p < 0.001. ns: not significant, One‐way ANOVA test, Dunnett's multiple comparisons test in comparison to the control.

The biosafety of SSA NBs is a critical parameter. The in vivo biosafety of SSA NBs was evaluated through blood parameter analysis in treated mice. Complete blood count analysis showed a decrease in white blood cell (WBC) levels in the SSA NBs group compared to other groups, indicating good wound healing in the mice. Other parameters, including red blood cells (RBC), hemoglobin (HGB), hematocrit (HCT), mean corpuscular volume (MCV), mean corpuscular hemoglobin (MCH), MCH concentration (MCHC), and mean platelet volume (MPV), were all within the normal range for mice (Figure 8A). Histopathological examination of major organs from different treatment groups, as shown in the H&E staining results (Figure 8B), revealed no significant tissue pathological damage. Compared to the control group, SSA NBs at therapeutic doses exhibited no toxic side effects and demonstrated excellent biocompatibility. Additionally, the safety of SSA NBs was evaluated using a hemolysis assay (Figure S11). The hemolysis rate of SSA NBs in various dispersion media was significantly below 5%, indicating good biocompatibility of SSA NBs.

To assess the in vivo biosafety of SSA NBs at low, medium, and high doses in comparison with Van, wound healing, bacterial clearance, and histological changes were systematically evaluated (Figure S12). SSA NBs promoted wound closure in a dose‐dependent manner, with the high‐dose group exhibiting the fastest healing, surpassing the Van group. Histological analyses (H&E and Masson staining) revealed more complete tissue regeneration and well‐organized collagen deposition in the high‐dose SSA NBs group, without evident necrosis or abnormal hyperplasia, indicating excellent tissue compatibility and regenerative potential. In contrast, the Van group showed slower wound healing, possibly due to potential inhibition of normal tissue repair or disturbance of the local microenvironment by Van. These findings suggest that SSA NBs, while exerting antibacterial effects, are more conducive to the physiological repair of skin wounds compared to Van. The levels of pro‐inflammatory cytokines (TNF‐α, IL‐6) decreased in a dose‐dependent manner in the SSA NBs groups, with the high‐dose group showing significantly lower levels than the Van group. This indicates that SSA NBs can effectively inhibit excessive inflammatory responses, avoiding tissue damage and delayed healing caused by chronic inflammation. Meanwhile, the repair‐related cytokine TGF‐β1 and anti‐inflammatory cytokine IL‐10 exhibited a reasonable expression pattern in the high‐dose group, promoting tissue remodeling while maintaining immune homeostasis. In comparison, the Van group had higher levels of pro‐inflammatory cytokines, which may trigger persistent local inflammation and affect healing quality. These results demonstrate that SSA NBs have favorable immunomodulatory activity and can synergistically regulate the inflammatory microenvironment during antibacterial processes, reflecting excellent biosafety. The antibacterial efficacy of SSA NBs increased with dose, and the high‐dose group had the lowest CFU count, comparable to or even better than that of the Van group. Importantly, unlike Van, SSA NBs did not induce obvious tissue toxicity or immune disorders while achieving high‐efficiency antibacterial effects. The dose‐dependent improvement in efficacy was accompanied by the maintenance of biocompatibility rather than an increase in toxicity. This characteristic originates from the material properties and mechanism of action of SSA NBs, which may achieve precise bacterial killing without extensive damage to host cells. In contrast, Van, as an antibiotic, may carry risks of dysbiosis or drug resistance. From the perspective of long‐term biosafety, SSA NBs show more promising application value.

## 3 Conclusion

Above all, this study presents and implements an innovative chemical self‐activating and self‐propelling APDT strategy aimed at simultaneously addressing two major challenges of MRSA biofilm infections: the physical barrier effect of the biofilm itself and the bacterial resistance defense mechanism. The key to this strategy lies in the chemical reaction between CPPO and locally accumulated H_2_O_2_ at the wound site. On one hand, it releases CO_2_ gas to provide propulsion for the SSA NBs nanosystem, promoting its deep penetration into the biofilm; on the other hand, the generated chemical luminescence intrinsically excites the loaded PS hypericin, inducing the production of bactericidal ROS. Results show that compared to using PS alone, SSA NBs not only significantly enhance the penetration ability into MRSA biofilms with a thickness of ≈15 μm but also cause complete destruction of the biofilm structure, with a clearance rate of over 90%. In addition to overcoming the physical barrier of the biofilm, SSA NBs can effectively overcome MRSA's intrinsic defense mechanism—hypericin inhibits CBS enzyme expression, cutting off the bacteria's self‐protective pathway of H_2_S production, while the continuous generation of ROS accelerates the bacteria's death process. It is worth noting that SSA NBs exhibit high killing efficiency against free MRSA cells and bacteria under biofilm states, making them widely applicable in actual wound environments. In summary, SSA NBs represent an efficient and safe localized therapy strategy for combating MRSA infections, providing a promising new approach for clinically eradicating MRSA‐infected wound biofilms. Its unique self‐activating and self‐propelling mode not only avoids the limitations of external light exposure but also endows the nanosystem with excellent biofilm penetration and dual bactericidal functions, offering new hope for the clinical treatment of refractory MRSA infections.

## 4 Experimental Section

### 4.1 Preparation of SSA NBs

SSA NBs were prepared via SSA using a one‐pot method. In brief, 3 mmol of CPPO was dissolved in 3 mL of anhydrous ethanol, while 1 mmol of Hypericin was dissolved in 1 mL of anhydrous ethanol. The mixture was sonicated until complete dissolution. Subsequently, CPPO was added dropwise into hypericin at a rate of 0.2 mL/min and stirred in the dark at 25°C for 3 h. After centrifugation at 13 000 rpm for 10 min, the supernatant was washed three times with distilled water and finally dispersed in 4 mL of distilled water.

### 4.2 Characterization of SSA NBs

Using TEM (JEM‐ARM300F, JEOL, Japan) and SEM (JSM‐7900F, JEOL, Japan) to observe the morphology of SSA NBs. The hydrodynamic size and polydispersity index (PDI) of SSA NBs were measured using a Malvern particle size analyzer (Nano ZS90, Malvern, UK). The characteristic absorption peaks of CPPO, hypericin, and SSA NBs were determined using a UV–vis near‐infrared spectrophotometer, while Fourier‐transform infrared spectroscopy (FTIR, Thermo Fisher Scientific, USA) was employed to collect infrared spectra using the potassium bromide pellet technique. After cleaning and fixing the sample, surface topography images were acquired using a calibrated AFM probe in tapping mode at an appropriate scan rate. Atomic fluorescence spectra were obtained using a fluorescence spectrophotometer (Hitachi, Japan, F‐7000).

### 4.3 Drug Loading Capacity of SSA NBs

In this experiment, we evaluated the content of CPPO and hypericin in SSA NBs using HPLC and UV–vis spectrophotometry. The CPPO content in SSA NBs was calculated by measuring the peak area under the same conditions where CPPO exhibited a peak at 235 nm at 5 min 35 s (mobile phase: water, acetonitrile). The drug loading capacity of hypericin in SSA NBs was determined by measuring the UV absorption at its characteristic wavelength of 592 nm.

### 4.4 Molecular Docking Simulation of SSA NBs

In order to investigate the formation mechanism of SSA NBs, we generated the structures of CPPO and hypericin using ChemDraw 3D and Gauss 5.0 software programs and performed molecular docking using Autodock. The interactions between CPPO and hypericin molecules in the complexes formed were further analyzed using the Multifwn software and the independent gradient model (IGM) method.

### 4.5 H_2_O_2_ Activation of SSA NBs to Produce CO_2_

Based on the ability of CPPO in SSA NBs to produce gas upon exposure to H_2_O_2_, we treated SSA NBs with 50 μM of H_2_O_2_, and observed the particle morphology under TEM after treatment for 5, 10, 30 min, and 2 h. Structural changes in SSA NBs after H_2_O_2_ treatment were analyzed using FTIR, and the production of 2‐hydroxy‐3,5,6‐trichlorobenzene carboxylate ester upon reaction of SSA NBs with H_2_O_2_ was analyzed using a UV–vis near‐infrared spectrophotometer. The motion trajectories of SSA NBs under different concentrations of H_2_O_2_ were observed using a nanoparticle motion tracking system, and the average motion velocity, average motion displacement, and MSD of the particles were calculated using the Video spot tracker with the formula

$$\mathrm{MSD}={\left(x_{\text{Δ}t}-x_{0}\right)}^{2}+{\left(y_{\text{Δ}t}-y_{0}\right)}^{2}$$

$x_{0}$: the x‐coordinate of the initial position of the particle; $x_{\text{Δ}t}$: the x‐coordinate of the particle after moving for a period of time; $y_{0}$: the y‐coordinate of the initial position of the particle; $y_{\text{Δ}t}$: the y‐coordinate of the particle after moving for a period of time

### 4.6 Calculation of HOMO‐LUMO Orbitals

The HOMO‐LUMO orbitals of CPPO and hypericin were calculated using Gaussian 09 software with the B3LYP method and 6‐31G(d) basis set.

### 4.7 Calculation of Reaction Gibbs Free Energy

In this study, quantum chemical calculations were performed using the Gaussian 09 software package. The B3LYP (Becke 3‐parameter exchange and Lee–Yang–Parr correlation) functional was employed, which is a widely used hybrid DFT method for molecular structure optimization and energy calculations. B3LYP combines the exchange energy from the Hartree–Fock method with the correlation energy from the local density approximation, providing a relatively accurate description of electronic structures. For the basis set, 6‐31G(d) was selected. This basis set is an extension of 6‐31G with added polarization functions (d‐functions) on valence electrons, which improves its performance in treating multiple bonds and noncovalent interactions. The 6‐31G(d) basis set is suitable for exploring molecular geometry optimization, energy calculations, and Gibbs free‐energy analysis. In practice, molecular geometry optimization was first carried out with Gaussian 09 to obtain a stable configuration. The optimized structures were then analyzed to confirm that they correspond to the lowest‐energy conformations.

### 4.8 Characterization of ROS Production in APDT

The production of ROS in APDT following activation by H_2_O_2_ was measured using the MB colorimetric method. Equal volumes (100 μL) of CPPO, hypericin, and SSA NBs were mixed with 100 μL of 10 mmol H_2_O_2_ solution and 100 μL of 10 mmol MB solution. The mixture was diluted to 1 mL with distilled water, incubated in the dark for 1 h, and the absorbance at 665 nm was measured. Similarly, the absorbance value at 665 nm (A) was compared to the baseline value (A_0_) (without addition of H_2_O_2_) to analyze ROS production in APDT for CPPO, hypericin, and SSA NBs under different concentrations of H_2_O_2_.

### 4.9 MRSA Cultivation

All antimicrobial experiments were conducted using MRSA. To obtain MRSA, individual colonies were inoculated into culture medium and then incubated at 37°C for 16–18 h until reaching the stable station. Subsequently, the culture was diluted with fresh TSB medium (4 mL) at a ratio of 1:100 and further grown at 37°C until reaching mid‐logarithmic phase (OD_600_ = 0.5).

### 4.10 Western Blot Analysis

The MRSA bacterial suspension treated with PBS, CPPO, hypericin, and SSA NBs was lysed using RIPA buffer. Protein concentration was normalized using the BCA protein assay kit. Proteins were separated using the SDS‐PAGE system (Bio‐Rad) and transferred onto PVDF membranes (Beyotime). PVDF membranes were blocked in protein‐free rapid blocking buffer for 30 min, followed by overnight incubation with primary antibodies. After washing with TBST buffer, membranes were incubated with secondary antibodies, and signal detection was performed using ECL substrate. Signals were collected and analyzed using the QuickChemi 5200 system (Monad).

### 4.11 H_2_S Signal Detection

Using a hydrogen sulfide (H_2_S) fluorescent probe to detect intracellular hydrogen sulfide in MRSA. Since bacterial suspensions cannot locally produce H_2_O_2_ to simulate the generation of H_2_S and ROS at wound sites, we added H_2_O_2_ to a concentration of 20 μM to detect the differences in related substances within MRSA. After treating MRSA planktonic cells with PBS, CPPO, hypericin, and SSA NBs, 20 μM H_2_O_2_ was added and incubated at 37°C for 1 h. The MRSA suspension was then centrifuged and washed with PBS, followed by a 20 min dark incubation with 1 μM WSP‐1 probe. After centrifugation, the free WSP‐1 was removed, and the cells were washed twice. A 4% formaldehyde solution was added for fixation. The prepared MRSA suspension was then placed on microscope slides and covered with glass coverslips. Finally, the fluorescence was observed using a Zeiss inverted fluorescence microscope.

### 4.12 Reactive Oxygen Species (ROS) Detection

For ROS detection, MRSA planktonic were treated with PBS, CPPO, hypericin, and SSA NBs, then treated with H_2_O_2_. Subsequently, the DCFH‐DA dye (10 μM) was added to the MRSA suspension and incubated in a light‐proof space for 20 min. After centrifugation to remove the fluorescent dye, the cells were fixed, mounted on slides, and observed using confocal laser scanning microscope (CLSM) to assess ROS fluorescence.

### 4.13 Minimum Inhibitory Concentrations (MICs)

The MICs of the control group, CPPO, hypericin, SSA NBs, and Van against MRSA planktonic cells were determined using the microdilution method.

### 4.14 Bacterial Killing Plate Assay

MRSA was collected and centrifuged with PBS (6000 rpm, 10 min) twice. Add 1.5 × 10^6^ CFU of MRSA bacterial colonies to each well of a 96‐well plate. MRSA suspension (50 μL) was coincubated with CPPO, hypericin, and SSA NBs for 3 h. A 10 000‐fold dilution was performed using PBS. For colony visualization, aliquots (20 μL) of the diluted bacterial solution were spread onto TSB agar plates and incubated overnight at 37°C.

### 4.15 Live/Dead Bacterial Staining

After treatment with PBS, CPPO, hypericin, and SSA NBs, 20 μM H_2_O_2_ was added to the MRSA suspension. The suspension was then centrifuged and dispersed in PBS, followed by a 15 min dark incubation with fluorescent dyes SYTO‐9 (2 μM) and PI (1 μM). The cells were washed twice with PBS. A 4% paraformaldehyde solution was added to fix and collect the MRSA. Finally, the observation was carried out using a laser scanning confocal microscope. All bacteria were stained green with SYTO‐9, while bacteria with compromised cytoplasmic membrane integrity were marked with red fluorescent PI.

### 4.16 Assessment of MRSA Using TEM

Following the above method, bacterial cultures were grown to mid‐logarithmic phase, after which aliquots were treated with the various formulations under investigation. Following centrifugation to collect the bacterial cells, the resulting pellets underwent two successive washing steps using PBS. followed by overnight agitation with 2.5% glutaraldehyde at 4°C Subsequently, negative staining was performed using phosphotungstic acid. A 10 μL drop was dried on a copper grid and observed under a TEM to assess the morphology of MRSA.

### 4.17 In Vitro Penetration of Deep Biofilms and Biofilm Viability Staining

The live/dead staining of the biofilm also utilizes SYTO‐9 and PI. Fluorescence imaging was performed using a CLSM. Since hypericin fluoresces under excitation at 592 nm, to observe the accumulation of biofilms and the penetration ability of SSA NBs, we also observed the fluorescence of hypericin. Z‐stack imaging through CLSM was employed to visualize the penetration of compounds/agents into the biofilm.

### 4.18 Crystal Violet Staining Method

MRSA biofilms were incubated separately with PBS, CPPO, hypericin, or SSA NBs at 37°C for 3 h. After treatment, the biofilms were stained with 0.1% crystal violet for 15 min and then rinsed thoroughly with distilled water to remove excess dye. Subsequently, 33% acetic acid was added to each well to dissolve the bound crystal violet. The absorbance at 560 nm was measured to quantify the biofilm biomass.

### 4.19 Observation of MRSA Membrane Structure Using Scanning Electron Microscopy

SEM was used to examine the membrane structure of MRSA within the biofilms. In brief, the MRSA biofilms, obtained by centrifugation and washing with PBS, were fixed overnight at 4°C with 2.5% glutaraldehyde. After incubation in 1% osmium tetroxide for 1 h, the samples were dehydrated in a series of graded ethanol solutions and tert‐butanol (50%, 75%, 90%, and 100%) for 10 min each. Subsequently, the samples were dried and observed using SEM.

### 4.20 Development of Mouse Skin Wound Infection Model

Male Balb/c mice (6–8 weeks old, 20–30 g) were obtained from Hangzhou Qizhen Experimental Animal Co., Ltd. The fur on their hind legs was removed using a depilatory device to develop the mouse skin wound infection model. Ensuring the skin was intact, the mice were administered pentobarbital intraperitoneally based on their body weight. Once fully anesthetized, a circular wound was created on the shaved area of the hind leg, avoiding the fascial layer. The mice were then randomly assigned to four groups (6 mice per group): the PBS control group (Group 1), the CPPO group (Group 2), the hypericin group (Group 3), and the SSA NBs group (Group 4). All animal experiments were approved by the Animal Experimental Research Center of Zhejiang Chinese Medical University and were conducted in accordance with the relevant guidelines and regulations. The approval number is IACUC‐20241125‐07.

### 4.21 Evaluation of In Vivo Antibiofilm Activity and Wound Healing Status

200 μL of PBS solution was applied to the wound. CPPO, hypericin, and SSA NBs (2 μg/mL, 200 μL) were separately dropped onto the wounds of the second, third, and fourth groups, respectively. Meanwhile, the body weight and wound size of the mice were measured on days 0, 1, 3, 5, 7, and 9 postmodeling under anesthesia. After 9 days of treatment, wound tissues from different groups of infections were collected, homogenized in sterile PBS, and quantitatively assessed for the elimination of MRSA biofilms using a plate counting method. On the 9th day of treatment, euthanasia was performed on some mice, followed by the dissection of skin tissues surrounding the infected wounds. Further evaluation included assessing residual MRSA bacteria, CBS enzyme activity, and the healing status of surrounding tissues.

### 4.22 Evaluation of In Vivo Anti‐Inflammatory Activity

On the 9th day, several cytokines associated with the inflammatory response, such as TNF‐α, TGF‐β, IL‐6, and IL‐10, were evaluated. To further assess the inflammatory response in the bloodstream, blood samples were collected on the 9th day. The expression levels of these pro‐inflammatory cytokines were measured using specific enzyme‐linked immunosorbent assay (ELISA) kits.

### 4.23 In Vivo Biosafety Assessment

To evaluate the biosafety of SSA NBs, the mice from each group were sacrificed at the end of the experiment. Major organs, including the heart, lungs, liver, spleen, and kidneys, were collected and processed into paraffin sections for pathological analysis. Specifically, the tissue samples were first fixed in 4% paraformaldehyde solution, followed by dehydration, paraffin embedding, and sectioning. The sections were then stained using the standard hematoxylin‐eosin (H&E) staining method. Additionally, on the 9th day of the experiment, blood samples were drawn from the mice for routine hematological testing to assess the potential systemic toxicity of the SSA NBs. Hemolysis Assay: Collect rabbit ear vein blood into anticoagulant tubes and prepare a 2% RBC suspension with saline. Set up a positive control (treated with 0.1% Triton X‐100) and a negative control (treated with PBS only). Add the remaining blood to the PBS dispersions of CPPO, hypericin, and SSA NBs. Incubate at 37°C for 1 h, then centrifuge at 1500 rpm for 10 min. Measure the absorbance of the supernatant at 540 nm using a microplate reader and calculate the hemolysis rate. The calculation formula is as follows

$$\mathrm{Hemolysis}\mathrm{rate}(\%)=\frac{{\mathrm{Abs}}_{\text{sample}}-{\mathrm{Abs}}_{\text{negative}}}{{\mathrm{Abs}}_{\text{positive}}-{\mathrm{Abs}}_{\text{negative}}}*100\%$$

### 4.24 In Vivo Dose‐Dependence Evaluation

Van was used as a positive control, and wound administration of low (10 μg/mL), medium (20 μg/mL), and high (40 μg/mL) doses was performed to evaluate bacterial eradication and tissue healing in mouse wounds, following the procedures described in sections Evaluation of In Vivo Anti‐inflammatory Activity and In Vivo Biosafety Assessment*.*

### 4.25 Statistical Analyzes

All experimental data were statistically analyzed using GraphPad Prism software (version 9, San Diego, CA, USA). Depending on the experimental design and the number of groups compared, appropriate statistical tests were selected: Student's t‐test for comparisons between two groups, and one‐way or two‐way ANOVA for multiple‐group comparisons. Quantitative results are presented as mean ± standard deviation (SD). A *p* value less than 0.05 was considered statistically significant. To ensure data reliability, all in vitro characterizations and cell experiments were independently repeated at least three times, and animal experiments were conducted with six parallel samples per group. These details have been added to the revised manuscript and highlighted accordingly.

## Supporting Information

Additional supporting information can be found online in the Supporting Information section. **Supporting Fig. S1:** Height profile of the supramolecular nanobowl measured along the indicated line in the AFM image. **Supporting Fig. S2:** FT‐IR spectra of CPPO, Hypericin, and SSA NBs. **Supporting Fig. S3:** The standard curve obtained from the peak areas of CPPO by HPLC and The standard curve obtained from the characteristic UV absorption of Hypericin. **Supporting Fig. S4:** The motion trajectories of SSA NBs under the action of 20 μM H_2_O_2_, along with the corresponding displacement, frequency, and calculated distance. **Supporting Fig. S5**: The motion trajectories of SSA NBs under the action of 10 μM H_2_O_2_, along with the corresponding displacement, frequency, and calculated distance. **Supporting Fig. S6:** The motion trajectories of SSA NBs under the action of 5 μM H_2_O_2_, along with the corresponding displacement, frequency, and calculated distance. **Supporting Fig. S7:** The motion trajectories of SSA NBs under the action of no H_2_O_2_, along with the corresponding displacement, frequency, and calculated distance. **Supporting Fig. S8:** Gibbs free‐energy profile for SSA‐NBs‐mediated PDT after H_2_O_2_ activation. **Supporting Fig. S9:** Schematic Diagram of Molecular Docking between CPPO and CBS Enzyme. **Supporting Fig. S10:** SSA NBs’ inhibitory effect against *E. coli* and *P. aeruginosa.* **Supporting Fig. S11:** Hemolysis Assay of SSA NBs in Different Dispersion Media. **Supporting Fig. S12:** In‐vivo efficacy studies of low, medium, and high‐dose SSA NBs versus vancomycin.

## Author Contributions

**WeiYe Ren**: Writing – review & editing, writing – original draft, software, methodology. **WeiYi Cheng**: Formal analysis, data curation, methodology. **Li He**: Writing – original draft, software. **JingQuan Chen**: Formal analysis. **Liting He**: Toxicity assessment. **Haorong Li**: Methodological investigation. **Guoying Zhou**: Experimental supervision & data processing. **Yinghui Wei**: Writing – review & editing, project administration, funding acquisition. **Ji‐Gang Piao**: Writing – review & editing, project administration, funding acquisition. **Dandan Bao**: Writing – review & editing, funding acquisition.

## Funding

This study was supported by National Key Research and Development Program of China (2023YFD1800102), Natural Science Foundation of Zhejiang Province (LY23H300002). The Research Project of Zhejiang Chinese Medical University (2025JKZKTS42, 2024JKZKTS24, 2024FSYYZQ31, 2025JKZDZC07), China University Industry‐University‐Research Innovation Fund (2024hr017), Zhejiang Provincial Traditional Chinese Medicine Science and Technology Program Project (2026ZL0298), the Hangzhou Key Natural Science Foundation Project (2025SZRJJ1206)

## Conflicts of lnterest

The authors declare no conflicts of interest.

## Supporting information

Supplementary Material

## Data Availability

The data that support the findings of this study are available from the corresponding author upon reasonable request.
