# Supplementary material for: A Chemiluminescence‐Activated Photodynamic Platform to Penetrate MRSA Biofilms and Counteract H2S‐Mediated Defense
Source: Small Sci. 2026 Mar 13;6(3):e202500618. doi: 10.1002/smsc.202500618 (PMC13098083; doi:10.1002/smsc.202500618)
Supplement: Supplementary file 1 — Supplementary Material [file SMSC-6-e202500618-s001.pdf]

A Chemiluminescence-Activated Photodynamic Platform to Penetrate MRSA Biofilms and Counteract H_2S-Mediated Defense

WeiYe Ren [b,1], *WeiYi Cheng* [b,1], *Li He* [b,1] , *JingQuan Chen* [b], *Liting He* [b], *Haorong Li* [e,*], *Guoying Zhou*[d,*] , *Yinghui Wei* [b,c,*], *Ji-Gang Piao* [b,*], *Dandan Bao* [a,*],

Supplementary Figures

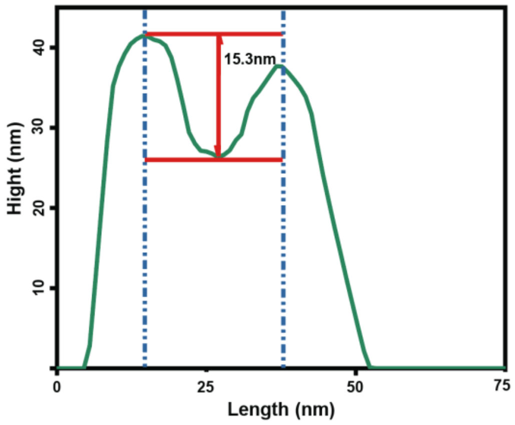

Figure S1. Height profile of the supramolecular nanobowl measured along the indicated line in the AFM image.

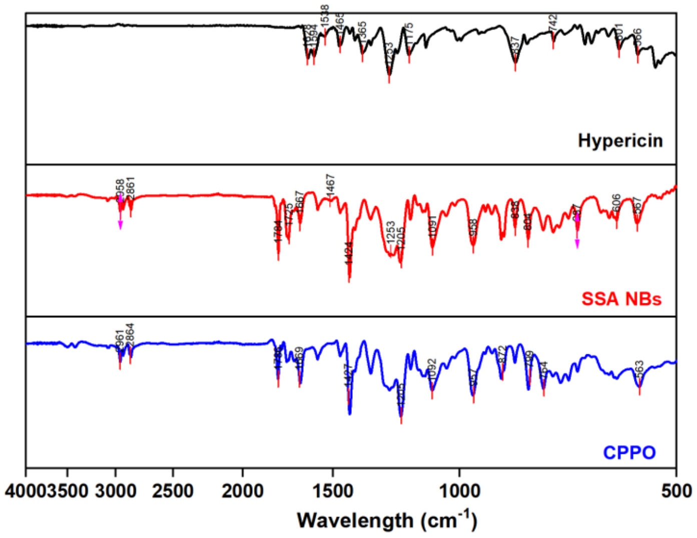

Figure S2. FT-IR spectra of CPPO, Hypericin, and SSA NBs.

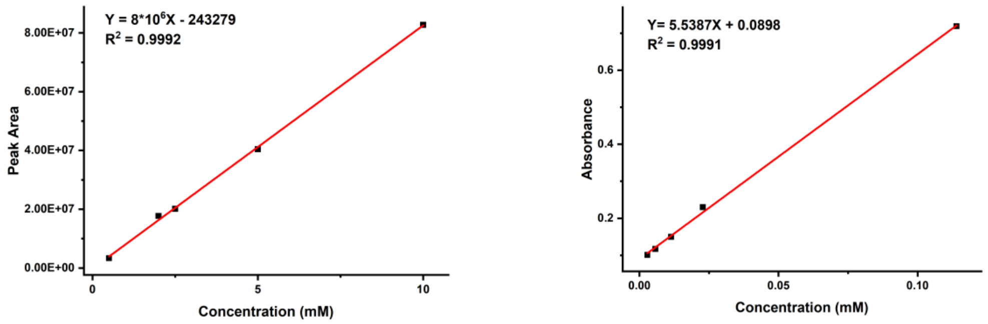

Figure S3. The standard curve obtained from the peak areas of CPPO by HPLC and The standard curve obtained from the characteristic UV absorption of Hypericin.

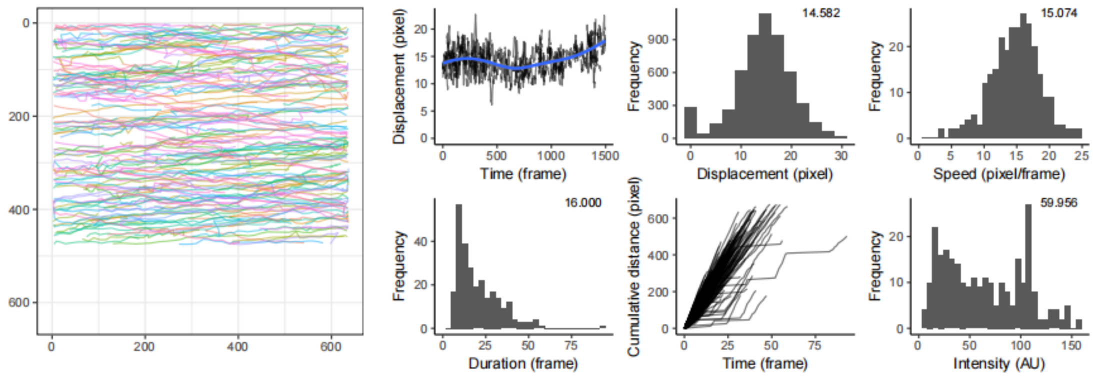

Figure S4. The motion trajectories of SSA NBs under the action of 20 μM H_2O_2, along with the corresponding displacement, frequency, and calculated distance.

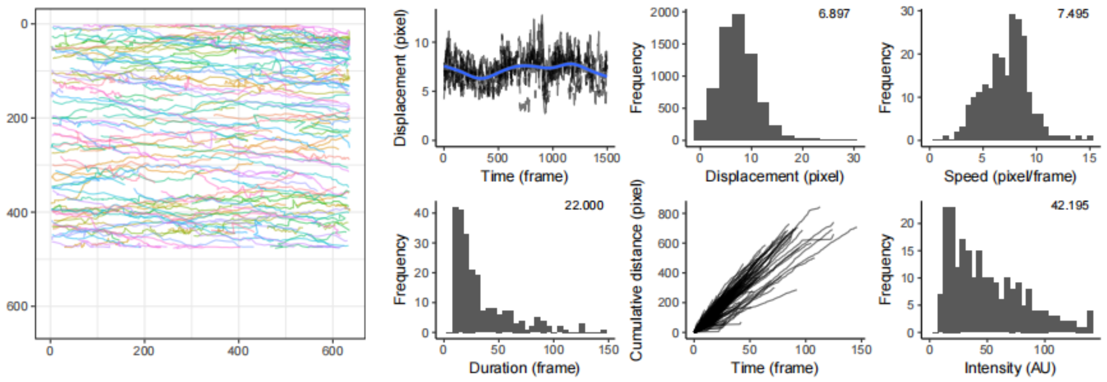

Figure S5. The motion trajectories of SSA NBs under the action of 10 μM H_2O_2, along with the corresponding displacement, frequency, and calculated distance.

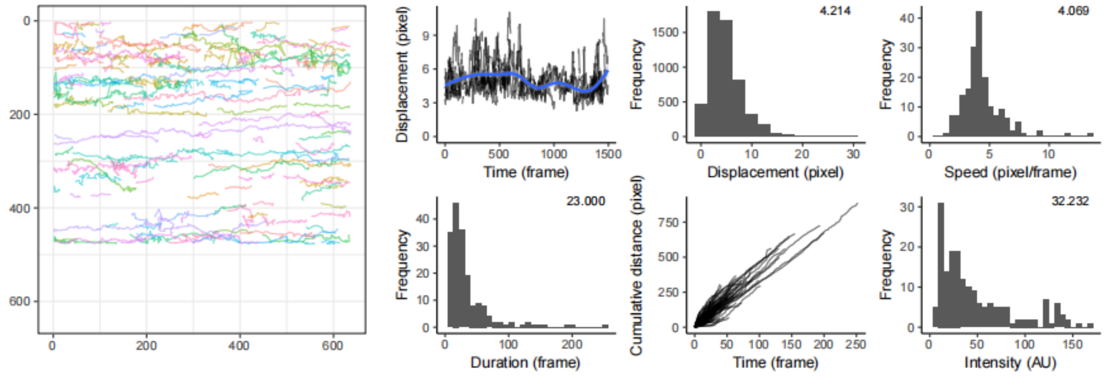

Figure S6. The motion trajectories of SSA NBs under the action of 5 μM H_2O_2, along with the corresponding displacement, frequency, and calculated distance.

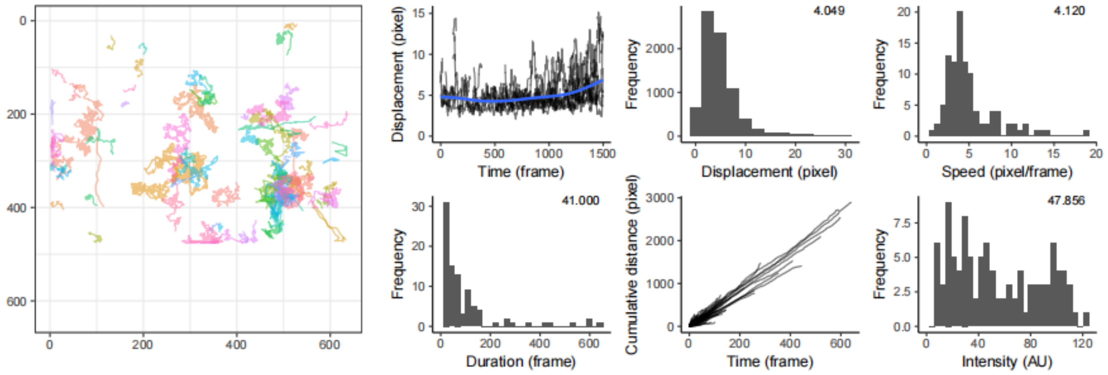

Figure S7. The motion trajectories of SSA NBs under the action of no H_2O_2, along with the corresponding displacement, frequency, and calculated distance.

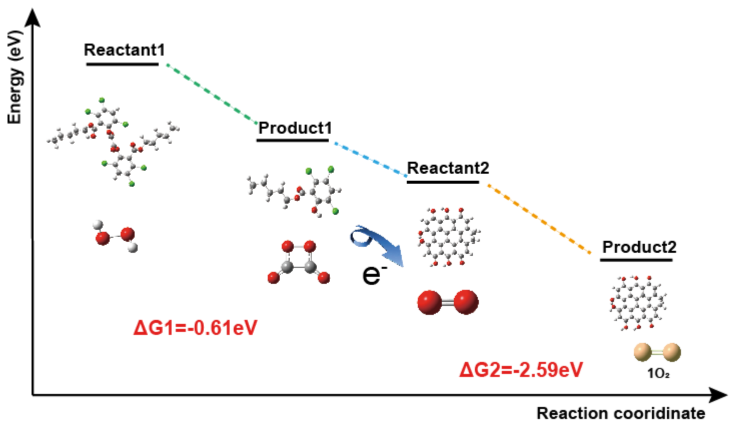

Figure S8. Gibbs free-energy profile for SSA-NBs-mediated PDT after H_2O_2 activation

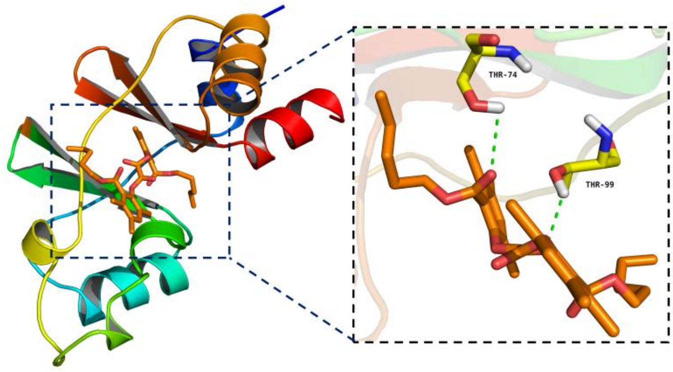

Figure S9. Schematic Diagram of Molecular Docking between CPPO and CBS Enzyme

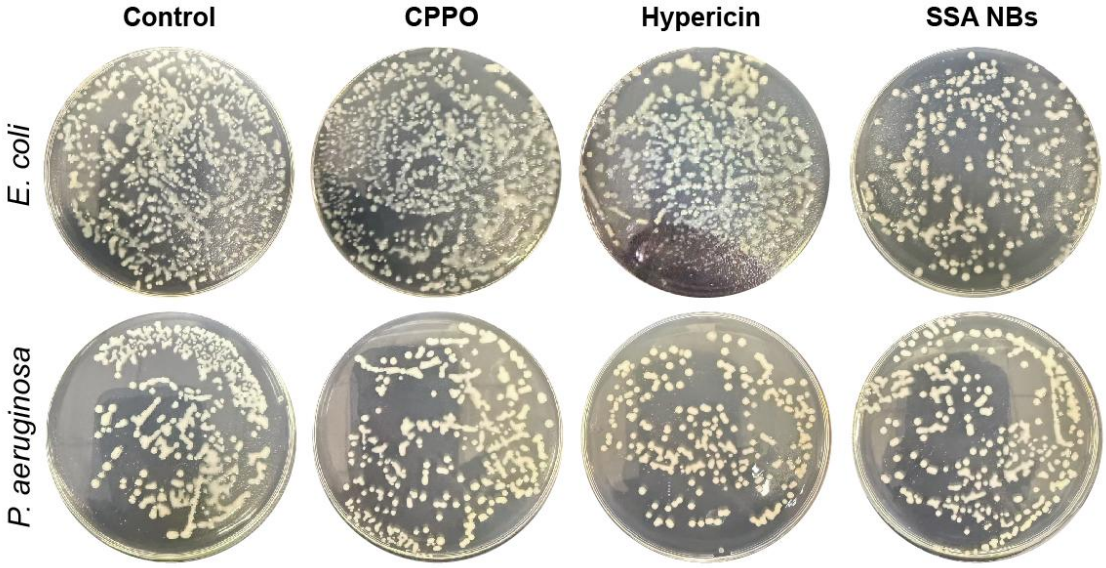

Figure S10. SSA NBs' inhibitory effect against *E. coli* and *P. aeruginosa.*

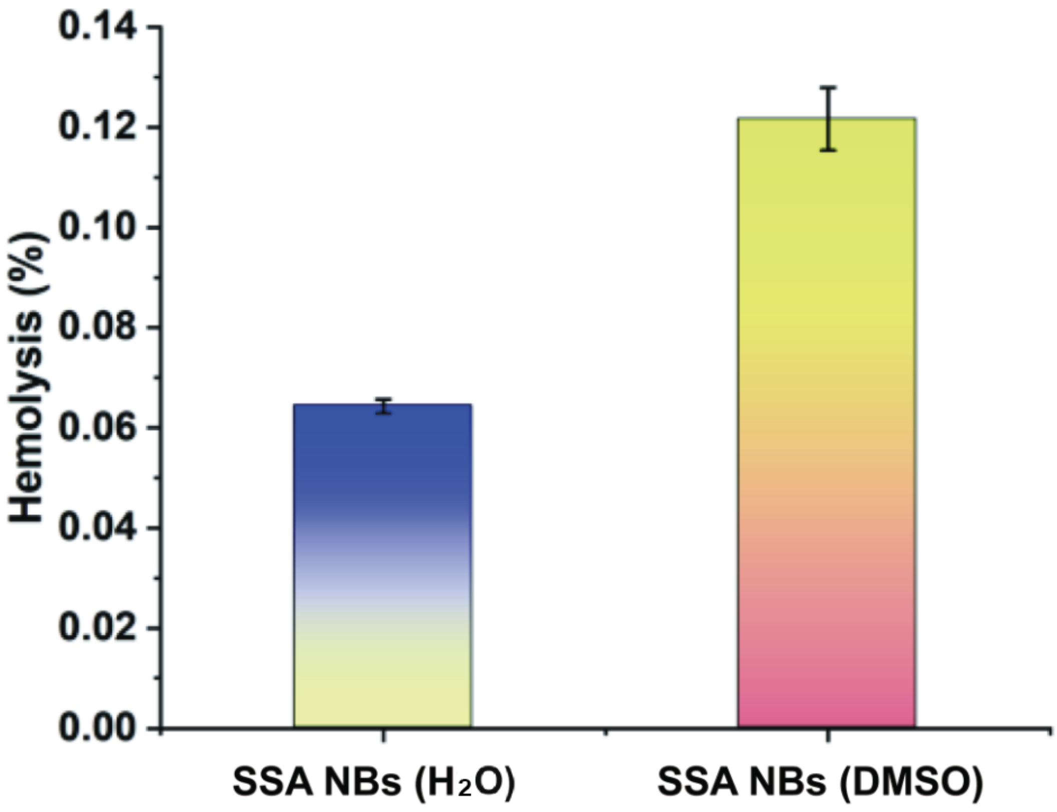

Figure S11. Hemolysis Assay of SSA NBs in Different Dispersion Media

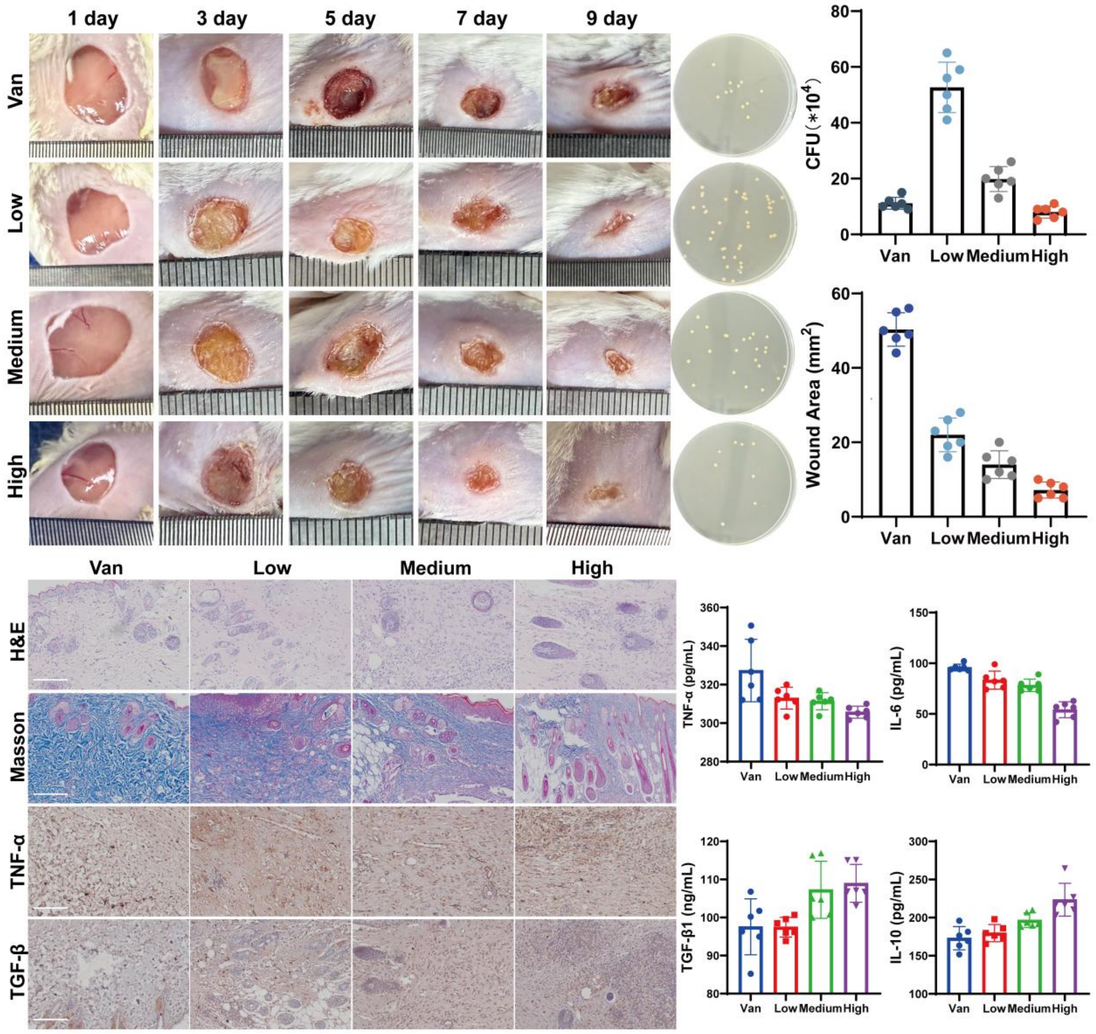

Figure S12. In-vivo efficacy studies of low, medium, and high-dose SSA NBs versus vancomycin.
